# Genome-level homology and phylogeny of Vibrionaceae (Gammaproteobacteria: Vibrionales) with three new complete genome sequences

**DOI:** 10.1186/1471-2180-13-80

**Published:** 2013-04-11

**Authors:** Rebecca B Dikow, William Leo Smith

**Affiliations:** 1 Committee on Evolutionary Biology, The University of Chicago, 1025 E. 57th St. Culver Hall 402, Chicago, IL 60637, USA; 2 Division of Fishes, The Field Museum, , 1400 S. Lake Shore Drive, Chicago, IL 60605, USA; 3 Present address: Center for Conservation and Evolutionary Genetics, National Zoological Park and Division of Mammals, National Museum of Natural History, Smithsonian Institution, PO Box 37012, MRC 108, Washington, DC 20013-7012, USA

## Abstract

**Background:**

Phylogenetic hypotheses based on complete genome data are presented for the Gammaproteobacteria family Vibrionaceae. Two taxon samplings are presented: one including all those taxa for which the genome sequences are complete in terms of arrangement (chromosomal location of fragments; 19 taxa) and one for which the genome sequences contain multiple contigs (44 taxa). Analyses are presented under the Maximum Parsimony and Maximum Likelihood optimality criteria for total evidence datasets, the two chromosomes separately, and individual analyses of locally collinear blocks. Three of the genomes included in the 44 taxon dataset, those of *Vibrio gazogenes*, *Salinivibrio costicola*, and *Aliivibrio logei* have been newly sequenced and their genome sequences are documented here.

**Results:**

Phylogenetic results for the 19-taxon datasets show similar levels of collinear subset of dataset incongruence as a previous study of 22 taxa from the sister family Shewanellaceae, while also echoing the strong phylogenetic performance of random subsets of data also shown in this study. Phylogenetic results for both the 19-taxon and 44-taxon datasets corroborate previous hypotheses about the placement of *Photobacterium* and *Aliivibrio* within Vibrionaceae and also highlight problems with how *Photobacterium* is delimited and indicate that it likely should be dissolved into *Vibrio* to produce a phylogenetic taxonomy. The 19-taxon and 44-taxon trees based on the large chromosome are congruent for the majority of taxa that are present in both datasets. Analyses of the 44-taxon sampling based on the second, small chromosome are quite different from those based on the large chromosome, which is not surprising given the dramatically divergent nature of the small chromosome and the difficulty in postulating primary homologies.

**Conclusions:**

The phylogenetic analyses presented here represent the most comprehensive genome-level phylogenetic analyses in terms of taxa and data. Based on the availability of genome data for many bacterial species on GenBank, many other bacterial groups would also be amenable to similar genome-scale phylogenetic analyses even when present in multiple contigs. The result that collinear subsets of data are incongruent with the concatenated dataset and with each other while random data subsets show very little incongruence echoes the result of previous work on Shewanellaceae. The 44-taxon phylogenetic analysis presented here thus represents the future of phylogenomic analyses in scope and complexity.

## Background

Members of Vibrionaceae (Gammaproteobacteria: Vibrionales) have been known since 1854 (Pacini) and were shown to be distinct much before pulsed-field gel electrophoresis revealed the most distinct diagnostic “morphological” feature, the existence of two chromosomes [1]. The interest in these bacteria is not surprising given that several species are pathogenic to humans and marine organisms and others are bioluminescent symbionts of marine fishes and squids *e.g.*[2-7]. Some lesser-known species are psychrophiles (live in cold temperatures), piezophiles (live at high pressures), or halophiles (live at high NaCl concentration; [8]). The diversity of ecologies represented by members of Vibrionaceae has led to enthusiastic genome sequencing in the group, which has focused most heavily on pathogenic species (more than 31 strains of *V. cholerae* are available on GenBank as of 2012).

A phylogenetic hypothesis based on complete genomes was desired for Vibrionaceae. While the analysis presented in [9] for Vibrionaceae was the most comprehensive to date (eight gene loci for 95 Vibrionaceae species) and provided the a hypothesis for a phylogenetic taxonomy for the group, the number of genomes already sequenced for Vibrionaceae lends itself to a genome-level analysis. While the specter of horizontal gene transfer always looms over phylogenetic analyses of bacteria, genome-level analyses take a proactive stance in the hopes of recognizing and quantifying problematic data partitions without blind dismissal of all phylogenetic signal. Because members of Vibrionaceae have two chromosomes, as discussed below, the genome-level phylogenetic analyses presented here provide phylogenetic evidence for the evolutionary scenarios that have been postulated for the maintenance of these two separate chromosomes.

There are also many Vibrionaceae species that are present on GenBank as multiple contigs. This was not the case for members of Shewanellaceae, the sister taxon to Vibrionaceae, for which a genome-level phylogenetic hypothesis was presented in [10]. It was the goal of this study to be able to include those taxa for which multiple contigs exist not only in order to produce the most well-sampled phylogenetic hypothesis possible, but also as an example of how genome-level phylogenetic analyses can be useful even when only incomplete genomes exist. Given that perfectly complete genome sequences are rare and as the price for genome sequencing decreases, there are likely to be more and more species sequenced by those interested in the allure of new datasets rather than the complete genome *per se*. As eukaryote taxa begin to be included in truly genome-level analyses (as distinct from simply mining genomes for individual genes and loci), there are also likely to be more missing data and parts of genomes that cannot necessarily be easily compared and homologized (*e.g.* junk DNA; although this has yet to be determined if it is indeed problematic). The 44-taxon phylogenetic analysis presented here thus represents the future of phylogenomic analyses in scope and complexity.

The presence of two chromosomes in all species Vibrionaceae has been of interest and investigated by many workers, but the origin and purpose of the second, smaller chromosome is subject to speculation *e.g.*[11]. While the total number of genes for species of Vibrionaceae is very similar to the total number of genes for those related bacteria with a single chromosome (*e.g.* Shewanellaceae), the second chromosome is not of similar composition to the first chromosome. It is smaller and more size variable [1]. It is considered a chromosome and not a plasmid, however. Chromosomes are distinguished from plasmids by the presence of “essential” genes required under all circumstances (*i.e.* not only when certain stresses are present) and in that the timing of replication of chromosomes occurs once per cell cycle while plasmids could possibly replicate more than once during a cell cycle or not at all [12]. When the first Vibrionaceae (*Vibrio cholerae*) genome sequence was completed [11], there were found to be few “housekeeping” and mostly “hypothetical” genes present on the small chromosome compared to the larger chromosome. From this, the authors hypothesized that absorption and expansion of an unrelated plasmid was the most likely source of the small chromosome.

*Vibrio gazogenes*, *Salinivibrio costicola*, and *Aliivibrio logei* were chosen as candidates for genome sequencing because the bulk of previous genome sequencing has focused on pathogenic species and strains. While *Vibrio gazogenes* has been classified in the genus *Vibrio* and yet in previous study of the Vibrionaceae family [9], it was placed within *Photobacterium*. There is little else in the literature regarding its phylogenetic placement, so it seemed to be a good candidate for genome sequencing. It is generally found in salt marshes and other marshy areas and produces red-pigmented colonies [13]. *Salinivibrio costicola*, is part of a clade of lesser-known species of Vibrionaceae, which also includes the species that were members of *Enterovibrio* and *Grimontia*. In the previous study mentioned above, these three genera were fount to be a clade it is found nested within a larger *Vibrio* clade [9]. This clade contains the halophilic extremophiles, none of which were represented as genome sequences on GenBank. *Aliivibrio logei*, formerly *Vibrio logei* and *Photobacterium logei*, is the predominant light-organ symbiont of squids in the genus *Sepiola*[14]. This species was chosen for genome sequencing as a next step in the attempt to complete sequencing of all bioluminescent species of *Vibrio* and *Photobacterium*.

## Results and discussion

### 19–taxon dataset

#### Results

**Figure 1:**
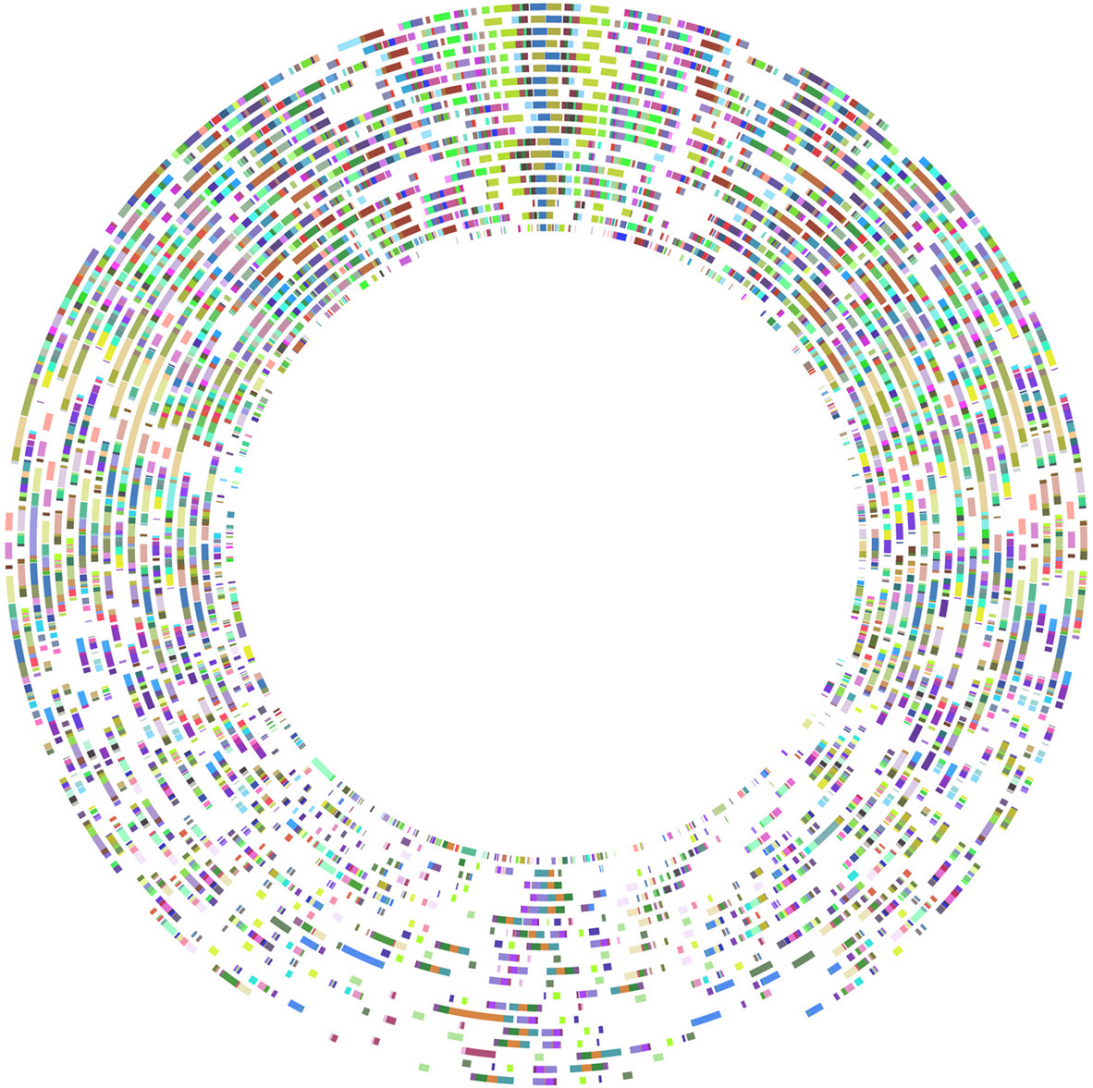
**Vibrionaceae large chromosome 306 LCB Circular Plot.** Circular 306 LCB plot for the Vibrionaceae large chromosome. Each circle represents a genome. From the innermost circle: *S. oneidensis*, *P. profundum*, *A. salmonicida*, *A. fischeri* ES, *A. fischeri* MJ, *V. anguillarum*, *V. furnissii*, *V. cholerae* 0395, *V. cholerae* M66, *V. cholerae* MJ, *V. cholerae* El Tor, *V. splendidus*, *V. vulnificus* YJ016, *V. vulnificus* M06, *V. vulnificus* CMC, *V. campbellii*, *V.* sp. EJY3, *V.* sp. Ex25, *V. parahaemolyticus*.

**Figure 2:**
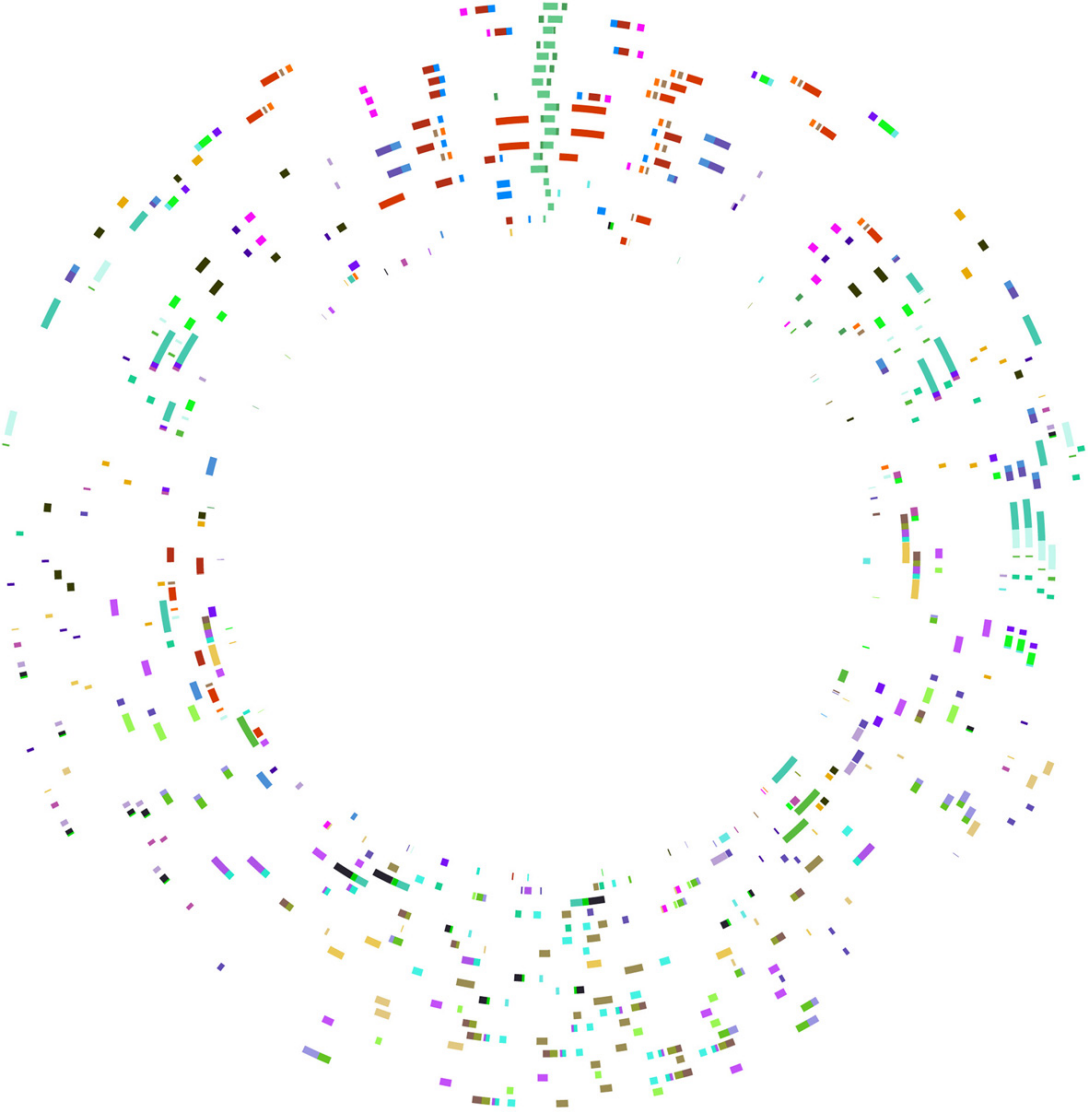
**Vibrionaceae small chromosome 37 LCB Circular Plot.** Circular 37 LCB plot for the Vibrionaceae small chromosome. Each circle represents a genome. From the innermost circle: *S. oneidensis*, *P. profundum*, *A. salmonicida*, *A. fischeri* ES, *A. fischeri* MJ, *V. anguillarum*, *V. furnissii*, *V. cholerae* 0395, *V. cholerae* M66, *V. cholerae* MJ, *V. cholerae* El Tor, *V. splendidus*, *V. vulnificus* YJ016, *V. vulnificus* M06, *V. vulnificus* CMC, *V. campbellii*, *V.* sp. EJY3, *V.* sp. Ex25, *V. parahaemolyticus*.

Table 1 contains the taxon details (strain names and numbers) and the GenBank accession numbers for the 19 taxa included in this dataset. Those taxa for which only one strain is included will be referred to by only their species name. Those taxa for which more than one strain is included will be referred to by species + strain name, abbreviated in most cases for the sake of brevity. The full names are listed in Table 1. For the large chromosome, 306 locally collinear segments of DNA (locally collinear blocks; LCBs) were found common to all taxa. For the small chromosome, 37 LCBs were found common to all taxa. The lengths of the alignments were, for the large chromosome, 3,644,395 bp and for the small chromosome, 426,592 bp. The lengths of individual LCB alignments for each chromosome are given in Additional file 1: Table S1 and Additional file 2: Table S2. It is striking that the small chromosome yielded so few LCBs. Even though it is the smaller chromosome, as a percentage, much less of this genome was able to be homologized. For example, for *V. cholerae* 0395, 140,579 bp out of 1,108,250 bp (12.7%) of the small chromosome was homologized. In contrast, 1,904,555 bp out of 3,024,069 (63%) of the large chromosome of *V. cholerae* was homologized. These measurements were made when gaps were removed from the alignments. In comparison to [10], 1,525,080 bp out of 4,969,803 bp (30.7%) of *Shewanella oneidensis* was able to be homologized using Mauve. Figure 1 shows the large chromosome LCBs plotted in circular form showing their arrangement in CGView. Each circle represents a genome in the analysis, and each colored block, an LCB. LCBs of the same color are putatively homologous. The orientation of taxa is based on the phylogenetic relationships presented below. Figure 2 shows the circular orientation of LCBs for the small chromosome. The individual genome circles have been rotated to maximize the visual similarity or orientation.

**Table 1:** Vibrionaceae taxon table: 19-taxon dataset

**Taxon name**	**Taxon #**	**Genbank accession numbers**	**Large chromosome**	**Small chromosome**
*Aliivibrio fischeri* ES114	14	NC_006840.2, NC_006841.2	2,897,536	1,330,333
*Aliivibrio fischeri* MJ11	15	NC_011184.1, NC_011186.1	2,905,029	1,418,848
*Photobacterium profundum* SS9	17	NC_006370.1, NC_006371.1	4,085,304	2,237,943
*Aliivibrio salmonicida* LFI1238	16	NC_011312.1, NC_011313.1	3,325,165	1,206,461
*Shewanella oneidnesis* MR-1T	18	NC_004347.1	4,969,803	
*Vibrio anguillarum* 775	13	NC_015633.1, NC_015637.1	3,063,913	988,135
*Vibrio cholerae* 01 biovar El Tor str. N16961	1	NC_002505.1, NC_002506.1	2,961,149	1,072,315
*Vibrio cholerae* 0395	0	NC_012582.1, NC_012583.1	3,024,069	1,108,250
*Vibrio cholerae* M66–2	2	NC_012578.1, NC_012580.1	2,892,523	1,046,382
*Vibrio cholerae* MJ-1236	3	NC_012668.1, NC_012667.1	3,149,584	1,086,784
*Vibrio* sp. EJY3	11	NC_016613.1, NC_016614.1	3,478,307	1,974,339
*Vibrio* sp. Ex25	6	NC_013456.1, NC_013457.1	3,259,580	1,829,445
*Vibrio furnissii* NCTC 11218	4	NC_016602.1, NC_016628.1	3,294,546	1,621,862
*Vibrio campbellii* ATCC BAA-1116	5	NC_009783.1, NC_009784.1	3,765,351	2,204,018
*Vibrio parahaemolyticus* RIMD 2210633	7	NC_004603.1, NC_004605.1	3,288,558	1,877,212
*Vibrio splendidus* LGP32	12	NC_011753.2, NC_011744.2	3,299,303	1,675,515
*Vibrio vulnificus* CMCP6	9	NC_004459.3, NC_004460.2	3,281,866	1,844,830
*Vibrio vulnificus* MO6–24/O	8	NC_014965.1, NC_014966.1	3,194,232	1,813,536
*Vibrio vulnificus* YJ016	10	NC_005139.1, NC_005140.1	3,354,505	1,857,073

The individual LCB trees are also listed in Additional file 1: Table S1 (large chromosome) and Additional file 2: Table S2 (small chromosome). For the large chromosome, LCB 25 and LCB 232 have the same topology (TNT). In Garli, LCB 1 has the same topology as LCB 169, LCB 72 has the same topology as LCB 191, LCB 30 has the same topology as LCB 62, LCB 115 has the same topology as LCB 150, LCB 80 has the same topology as LCB 257, LCB 178 has the same topology as LCB 293. This means 331 out of 343 are unique. The tree resulting from the large chromosome LCBs concatenated (RaxML) is same as LCB 205 (Garli). All other topologies are unique, including when comparing among datasets and optimality criteria. Additional file 3: Table S3 shows the topologies generated when random subsets of data are selected with both TNT and ML (RaxML or Garli). These trees are largely congruent, with differences occurring in the placement *V. splendidus* in both chromosomes, in *P. profundum* in the small chromosome, and within species differences in the relationships among *V. cholerae* in the small chromosome and in one case a difference in the relationships among *V. vulnificus* strains.

**Figure 3:**
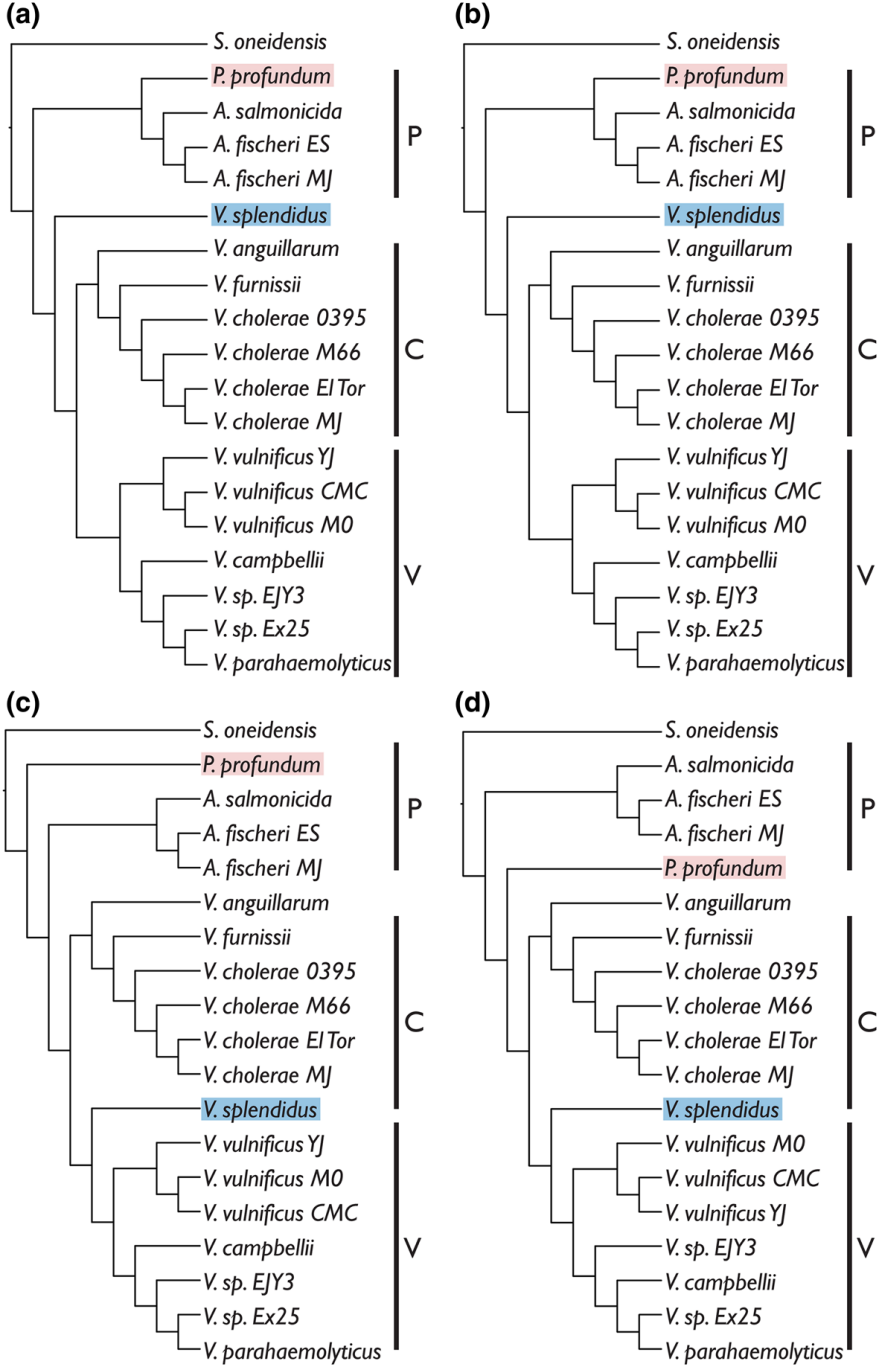
**Vibrionaceae 19–taxon trees from analysis of concatenated datasets.** Topologies resulting from analyses of concatenated 19–taxon datasets. (**a**) RaxML large chromosome, and both chromosomes concatenated, (**b**) RaxML small chromosome, (**c**) TNT large chromosome and both chromosomes concatenated, and (**d**) TNT small chromosome. Clades are labeled P=*Photobacterium* clade, C=*V. cholerae* clade, O=*V. orientalis* clade, and V=*V. vulnificus* clade.

**Figure 4:**
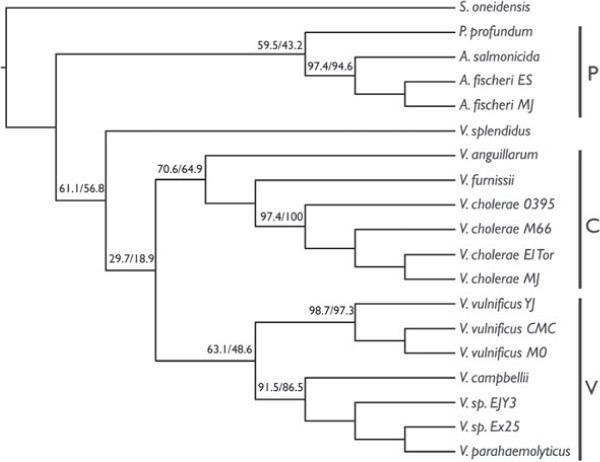
**Vibrionaceae 19–taxon RaxML tree with support values.** Topology resulting from a RaxML analysis of the large chromosome and also both chromosomes concatenated with support values at the nodes. The first number represents the percentage of LCBs of the large chromosome that when analyzed with ML, also contain that particular node. The second number represents the percentage of LCBs on the small chromosome that when analyzed with ML, also contain that particular node.

Figure 3 shows the topologies resulting from analyses of LCBs in concatenation from the large, small, and both chromosomes concatenated. Clades are labeled P=*Photobacterium* clade, C=*V. cholerae* clade, O=*V. orientalis* clade, and V=*V. vulnificus* clade. This will allow the easy tracking of common groups of species throughout the discussion. Figure 4 shows the topology resulting from analysis of the large chromosome in RaxML (this tree was the same as that when the small and large chromosomes were concatenated). Instead of bootstrap or jackknife support, which are 100% for all nodes when so many data are included, the percentage of LCBs from both the large and small chromosomes for which individual analysis also produced the node of interest is shown above the nodes. This could be considered a level of support when traditional methods do not provide any variation in levels across the tree. Trees resulting from random selection of nucleotides from concatenated alignments are shown in Additional file 4: Table S6. Data have been deposited on Dryad.

#### Discussion

*Shewanella oneidensis* is the only outgroup species included because Shewanellaceae is known to be sister to Vibrionaceae based on previous work [1] and because the inclusion of additional, more distant outgroup taxa would likely further reduce the percent coverage of LCBs present in all taxa, particularly since the number of ingroup taxa in this study was more than twice what it was in the recent study on Shewanellaceae [10]. In that paper, three outgroup species were chosen, of three different genera, because there was no phylogenetic precedent showing which genus would be an appropriate outgroup, or even if these outgroup genera were distinct from the ingroup genera in a phylogenetic sense. The % primary homology coverage is 29.4% (for *V. cholerae* 0395; when both chromosomes are added together) for the Vibrionaceae 19–taxon study and 30.7% for the Shewanellaceae study with (*S. oneidensis*) [10]. The number of included taxa is the most obvious contributor. It could also vary based on how that group is defined (*i.e.* a genus in one family might be much more variable than a genus in a different family) or depending on the evolutionary history of a particular group. The extreme divergence of the small chromosome of Vibrionaceae is likely part of their ability to occupy diverse ecological niches.

The results in terms of phylogenetic incongruence among datasets within the 19–taxon dataset are quite similar to those presented in [10] for Shewanellaceae in the pattern of unique trees for individual LCBs and a comparable number of LCBs of average size. For the individual LCB analyses, there was no overlap among optimality criteria in that none of the TNT topologies were the same as any Garli topologies. Two LCBs had the same topology in TNT and 12 had the same topology in Garli. This is a remarkably small number. There is strong congruence, however, between optimality criteria when we consider the analyses based on concatenation of LCBs. For ML, the large chromosome tree topology and the small chromosome tree topology differ only in the placement of *V. vulnificus* strains within the *V. vulnificus* clade. For MP, the large chromosome tree topology and the small chromosome tree topology also differ in the placement of *V. vulnificus* strains within the *V. vulnificus* clade and additionally, swap *V. sp. EJY3* and *V. campbellii*, and finally in the placement of *P. profundum*. The differing results between optimality criteria is interesting. In Figure 3, *P. profundum* has been highlighted with red and *V. splendidus* has been highlighted with blue to show how these taxa are placed differently in MP and ML. As mentioned in the introduction, *P. profundum* lives at high pressures and is not bioluminescent and both of these traits distinguish it from the rest of the *Photobacterium* species included here [8]. *Vibrio splendidus*, a pathogen of oysters (and other invertebrates; [15]) is placed at the base of either the C (*V. cholerae*) clade or the V (*V. vulnificus*) clade. In [9], *V. splendidus* was placed in a clade with nine other species that are not represented here (no complete genome sequences exist for these species). This might be why its placement is variable.

The trees produced by generating random subsets of data performed quite well in approximating the trees resulting from concatenation of LCBs (Additional file 4: Table S6). There was variation in the placement *V. splendidus* in both chromosomes, in *P. profundum* in the small chromosome along with a few instances of variation in within–species relationships. The uncertainty in placing *V. splendidus* and *P. profundum* is real and it is likely that only the addition of more taxa will solve this problem. In Figure 4, the ML tree for the large chromosome and both chromosomes concatenated is shown with “support” values at the nodes. Here, support was calculated by counting the number of individual LCB trees (ML; listed in Additional file 1: Table S1 and Additional file 2: Table S2) that also contained each node. As expected, the support for the *Photobacterium* + *Aliivibrio* clade is somewhat low; 59.5% of the individual LCBs analyzed contain that node for the large chromosome and 43.2% for the small chromosome. *P. profundum* is often placed at the base of the *Vibrio* clade instead of with the other species of *Photobacterium*. The non–monophyly of *Photobacterium* will be a theme continued below in discussion of the 44–taxon dataset. The node with the lowest support is that leading to the rest of *Vibrio* when *V. splendidus* is basal to the *Vibiro* clade. This is due to the variable placement of *V. splendidus*. The differences between optimality criteria in the concatenated dataset (Figure 3(a) and 3(c)) are also represented within optimality criterion when it comes to the individual LCB trees. The fact that the support values are somewhat low throughout the tree, underscores the fact that the individual LCB trees are different, and not just for one or two nodes.

### 44–taxon dataset

#### Results

**Figure 5:**
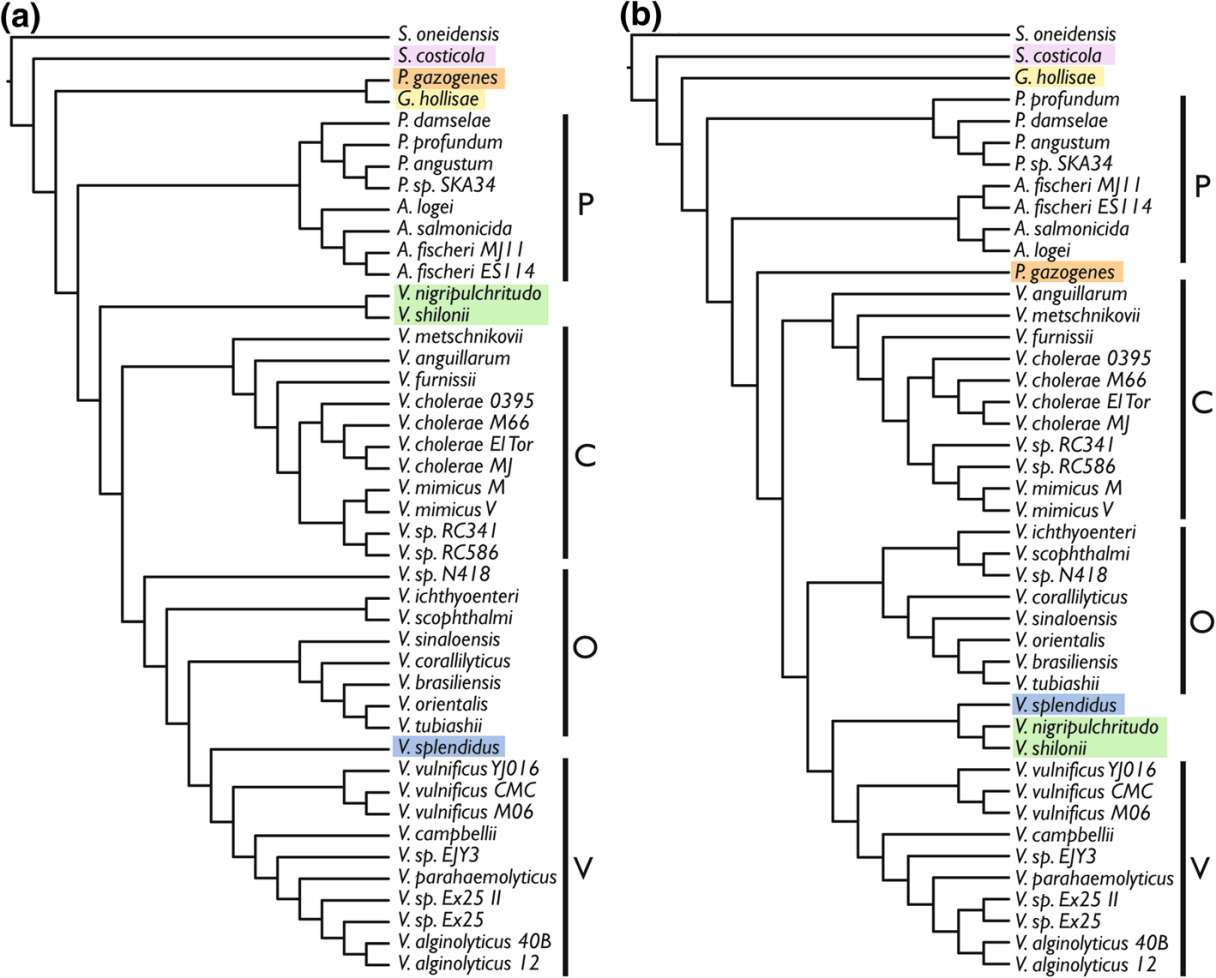
**Vibrionaceae Large Chromosome Trees: 44–Taxon Dataset.** Topologies resulting from analysis of the Vbirionaceae large chromosome for all 44 taxa: (**a**) TNT, (**b**) RaxML.

**Figure 6:**
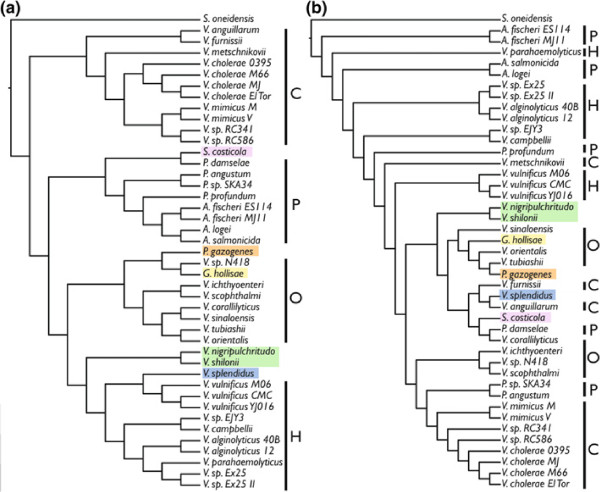
**Vibrionaceae small chromosome trees: 44–taxon dataset.** Topologies resulting from the analysis of the Vibrionaceae small chromosome for all 44 taxa: (**a**) TNT, (**b**) RaxML. Clades are labeled P=*Photobacterium* clade, C=*V. cholerae* clade, O=*V. orientalis* clade, and V=*V. vulnificus* clade.

Table 2 contains the taxon details (strain names and numbers) and the GenBank accession numbers for the 44 taxa included here (*V. brasiliensis* is excluded for the small chromosome) as well as the number of nucleotide base–pairs that were found to be primary homologs in Mauve for both the large and small chromosomes. Because of the way Mauve was run incrementally as described in the methods section to combat computational problems, only a single, large LCB resulted from each final analysis. The large chromosome produced an alignment with 26,557,925 bp and the small chromosome produced an alignment with 3,555,373 bp. The large chromosome trees for both TNT (gaps as fifth state) and RaxML are shown in Figure 5. As mentioned above, jackknife and bootstrap support values are uninformative when so many data are included. The large chromosome TNT tree has a length of 37,621,861 steps. The small chromosome trees for both TNT and RaxML are shown in Figure 6. The small chromosome TNT tree has a length of 4,014,864 steps.

**Table 2:** Vibrionaceae taxon table: 44–taxon dataset

**Taxon**	**Genbank accession numbers**	**Total length (bp)**	**MAUVE homologies (bp)**
*Aliivibrio fischeri* ES114	NC_006840.2, NC_006841.2	1,856,902	178,215
*Aliivibrio fischeri* MJ11	NC_011184.1, NC_011186.1	1,873,671	186,172
*Aliivibrio logei* ATCC 35077	PRJNA183872	806,834	174,234
*Aliivibrio salmonicida* LFI1238	NC_011312.1, NC_011313.1	1,899,286	169,047
*Grimontia hollisae* CIP 101886T	NZ_ADAQ00000000.1	780,144	3,571
*Photobacterium angustum* S14	NZ_AAOJ00000000.1	1,757,815	97,666
*Photobacterium damselae damselae* CIP 102761T	NZ_ADBS00000000.1	1,114,253	66,414
*Photobacterium profundum* SS9	NC_006370.1, NC_006371.1	1,877,292	115,879
*Photobacterium* sp. SKA34	NZ_AAOU00000000.1	1,688,145	58,021
*Salinivibrio costicola* ATCC 33505	PRJNA183873	720,523	60,549
*Shewanella oneidnesis* MR–1T	NC_004347.1	1,749,411	225,319
*Vibrio alginolyticus* 12	NZ_AAPS00000000.1	2,445,375	384,938
*Vibrio alginolyticus* 40B	NZ_ACZB00000000.1	2,446,712	325,598
*Vibrio anguillarum* 775	NC_015633.1, NC_015637.1	1,870,670	115,992
*Vibrio brasiliensis* LMG 20546	NZ_AEVS00000000.1	2,532,693	
*Vibrio cholerae* 01 biovar El Tor str. N16961	NC_002505.1, NC_002506.1	1,879,133	142,138
*Vibrio cholerae* 0395	NC_012582.1, NC_012583.1	1,904,555	140,579
*Vibrio cholerae* M66–2	NC_012578.1, NC_012580.1	1,870,580	142,049
*Vibrio cholerae* MJ–1236	NC_012668.1, NC_012667.1	2,003,477	142,071
*Vibrio corallilyticus* ATCC BAA–450T	NZ_ACZN00000000.1	3,063,355	622,314
*Vibrio furnissii* NCTC 11218	NC_016602.1, NC_016628.1	1,923,865	119,149
*Vibrio campbellii* ATCC BAA–1116	NC_009783.1, NC_009784.1	2,045,935	185,917
*Vibrio gazogenes*ATCC 43941	PRJNA183874	644,150	10,363
*Vibrio ichthyoenteri* ATCC 700023T	NZ_AFWF00000000.1	2,168,419	224,598
*Vibrio mediterranei* AK1	NZ_ABCH00000000.1	1,738,358	126,904
*Vibrio metschnikovii* CIP 69.14T	NZ_ACZO00000000.1	1,923,459	147,899
*Vibrio mimicus* MB451	NZ_ADAF00000000.1	2,166,746	457,366
*Vibrio mimicus* VM223	NZ_ADAJ00000000.1	2,194,901	442,251
*Vibrio nigripulchritudo* ATCC 27043T	NZ_AFWJ00000000.1	1,895,040	102,051
*Vibrio orientalis* CIP 102891T	NZ_ACZV00000000.1	2,328,799	336,533
*Vibrio parahaemolyticus* RIMD 2210633	NC_004603.1, NC_004605.1	1,956,217	182,533
*Vibrio scophthalmi* LMG 19158T	NZ_AFWE00000000.1	1,734,066	94,310
*Vibrio sinaloensis* DSM 21326	NZ_AEVT00000000.1	2,010,019	160,804
*Vibrio* sp. EJY3	NC_016613.1, NC_016614.1	1,960,726	148,390
*Vibrio* sp. Ex25	NC_013456.1, NC_013457.1	1,947,774	174,533
*Vibrio* sp. Ex25–2	NZ_AAKK00000000.2	1,935,036	156,969
*Vibrio* sp. N418	NZ_AFWD00000000.1	782,440	14,868
*Vibrio* sp. RC341	NZ_ACZT00000000.1	2,797,657	424,863
*Vibrio* sp. RC586	NZ_ADBD00000000.1	2,846,476	436,330
*Vibrio splendidus* LGP32	NC_011753.2, NC_011744.2	1,977,039	117,312
*Vibrio tubiashii* ATCC 19109T	NZ_AFWI00000000.1	2,359,746	318,328
*Vibrio vulnificus* CMCP6	NC_004459.3, NC_004460.2	1,954,971	116,837
*Vibrio vulnificus* MO6–24/O	NC_014965.1, NC_014966.1	2,008,045	165,578
*Vibrio vulnificus* YJ016	NC_005139.1, NC_005140.13	1,952,622	166,723

#### Discussion

The major Vibrionaceae clades represented here, P (=*Photobacterium*), C (=*V. cholerae*), O (=*V. orientalis*), and V (=*V. vulnificus*) are shown in Figure 5 as recovered by the MP and ML analyses of the large chromosome. There are minor differences among the members of the clades between MP and ML and in MP, “clade” O is rendered paraphyletic by *V.* sp. N418. The main topological differences occur in the placement of a few species. *Vibrio gazogenes*, which was also placed within *Photobacterium* in [9], is sister to *G. hollisae* here (MP; Figure 5(a) highlighted in orange) at the base of the entire tree (along with *S. costicola*) and at the base of the *Vibrio* clade in ML (Figure 5(b)). Sister species *V. nigripulchritudo* and *V. mediterranei* are placed at the base of the entire *Vibrio* clade in MP (Figure 5(a) highlighted in green) and in ML, at the base of clade V with *V. splendidus* (Figure 5(b)). *Vibrio splendidus* is also at the base of clade V in MP (Figure 5(a) highlighted in blue).

Beyond the differences between MP and ML, what is most interesting is the placement of *S. costicola* (pink), *G. hollisae* (yellow), and *V. gazogenes* (orange). The placement of these species at or near the base of the tree was a surprise. In [9], *G. hollisae* and *S. costicola* were both in a clade of extremophilic species deep within the larger *Vibrio* clade. The possibility of long branch attraction pulling them to the base here was investigated by removing each of these species one at a time and reanalyzing in TNT [16]. Each of these three species were always placed at the base, whether the other two taxa were present or not. All three also had the lowest % primary homology coverage for both the large and small chromosome (Table 2).

The small chromosome produced contrasting results when comparing MP to ML (Figure 6(a) and (b)). For MP, the 4 major clades were preserved, but the C and P clades swapped places, moving *Photobacterium* from its basal position and into *Vibrio*. *Salinivibrio costicola* was at the base of *Photobacterium* and *G. hollisae* and *V. gazogenes* were in the O clade. ML did not find any of the major clades to be monophyletic (Figure 6(b)). It was unexpected that the small chromosome would produce such differing results, especially since it did not do so in the 19–taxon analysis. There, the small chromosome topologies were largely congruent with the large chromosome topologies (Figure 3). The variation in size of the small chromosome is also present in the variation in % primary homology coverage by Mauve, where there was also large variability among taxa. Those taxa for which close relatives were also able to be included usually had a larger % coverage, which is expected given the way Mauve looks for primary homologies. Differences could also be present in the completeness of the genome sequences. Perhaps the small chromosome is the more difficult to assemble and the genomes that are present in multiple contigs are missing more of the small chromosome than the large. This might make the phylogenetic hypotheses suffer because of the lack of primary homology. This could explain why the 19–taxon small and large chromosome datasets result in a similar topologies, because they are based on completely assembled genomes.

### New genome sequences

#### Results

**Figure 7:**
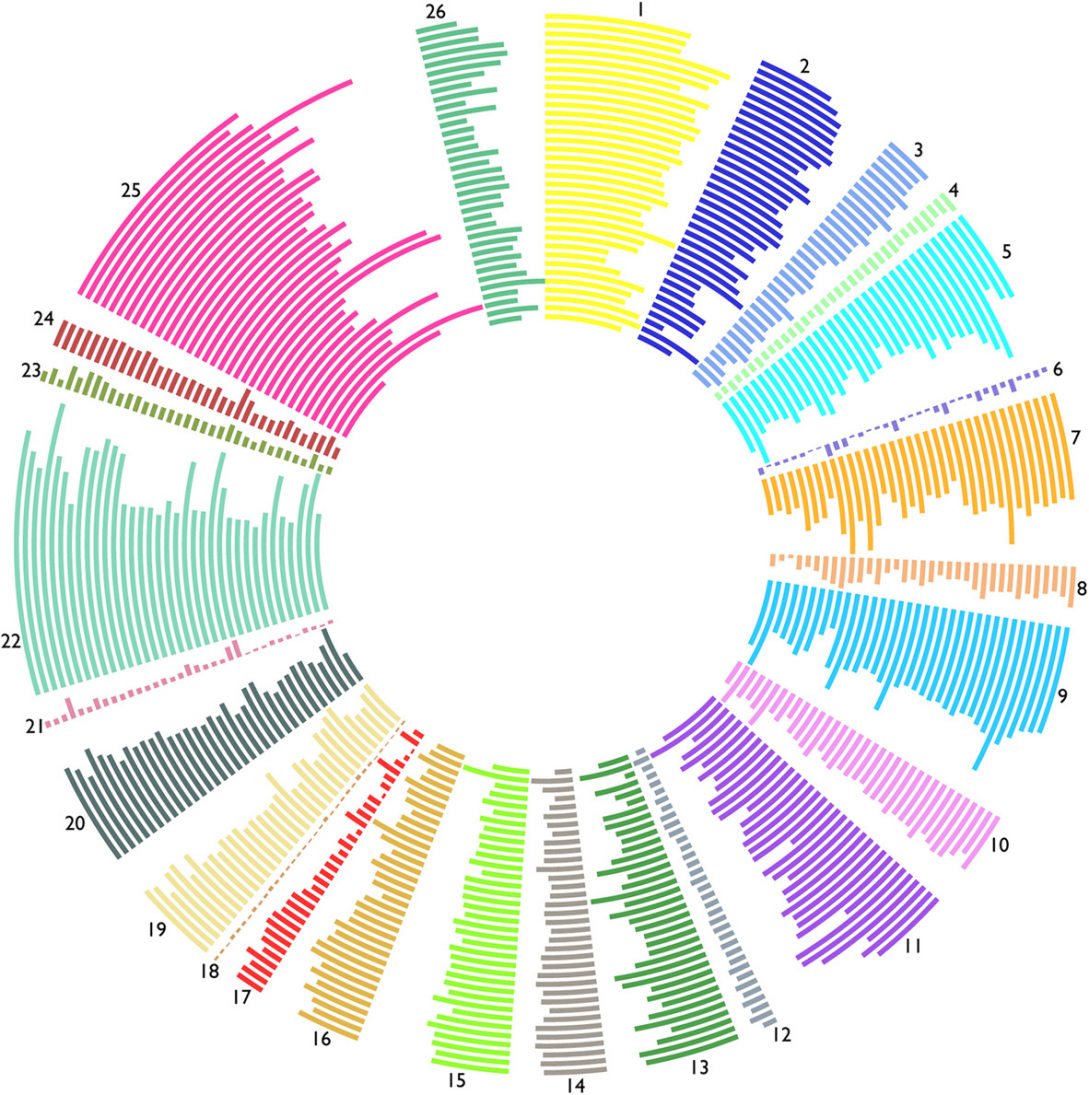
**RAST subsystems Circular Plot.** From inner to outer: *S. oneidensis*, *S. costicola*, *V. gazogenes*, *G. hollisae*, *P. damselae*, *P. profundum*, *P. angustum*, P. sp. SKA34, *A. logei*, *A. salmonicida*, *A. fischeri* ES114, *V. nigripulchritudo*, *V. mediterranei*, *V. metschnikovii*, *V. anguillarum*, *V. furnissii*, *V. cholerae* El Tor, *V. mimicus* M, *V.* sp. RC341, *V.* sp. RC586, *V.* sp. N418, *V. ichthyoenteri*, *V. scophthalmi*, *V. sinaloensis*, *V. corallillyticus*, *V. brasiliensis*, *V. orientalis*, *V. tubiashii*, *V. splendidus*, *V. vulnificus* CMC, *V. campbellii*, *V.* sp. EJY3, *V. parahaemolyticus*, *V.* sp. Ex25, *V. alginolyticus* 12.

For *S. costicola*, 61 contigs resulted, and the concatenated total length of the genome is 6,193,442 bp. For *A. logei*, 19 contigs resulted, and the concatenated total length of the genome is 5,424,165. For *V. gazogenes*, 36 contigs resulted, and the concatenated total length of the genome is 6,306,541 bp. These assemblies took 36 hours (approximately 250 computer hours) per 10 million sequences. Contigs have been submitted to GenBank (*numbers pending*). Annotations resulted in 5,575 coding sequences for *S. costicola*, 4,807 coding sequences for *A. logei*, and 5,616 coding sequences for *V. gazogenes*. The number of genes in all RAST subsystems as well as the number of tRNAs and coding sequences for all 35 species included in the 44–taxon dataset (a single strain was chosen for each species) are shown in Additional files 3: Table S3, Additional file 5: Table S4 and Additional file 6: Table S5. These data are also shown graphically in Figure 7 with the subsystem abbreviations shown in the tables.

#### Discussion

The gene content variation based on RAST subsystems across the 35 total species included in this taxon sampling provides another way to compare genomes (Additional files 3: Table S3, Additional file 5: Table S4 and Additional file 6: Table S5; Figure 7). The total number of coding sequences ranges from 3,404 (*V. metschnikovii*) to 5,700 (*V. nigripulchritudo*). There is a large variation in the number of tRNAs, from 57 (*V. sinaloensis*) to 223 (*P. damselae*). The *V. vulnificus* and *Photobacterium* group, some members of the *V. vulnificus* group, plus *G. hollisae* and *S. costicola* have the most tRNAs. These are the clades that contain bioluminescent taxa and *G. hollisae* and *S. costicola*, because they are placed at the base of *Photobacterium*, might actually be members of *Photobacterium*. Future work could include looking at the genes of particular subsystems and their representative presence in different LCBs and looking at those genes that are not assignable to subsystems to find genes that might be unique to Vibrionaceae.

## Conclusions

The placement of *V. gazogenes*, *S. costicola*, and *G. hollisae* at the base of Vibrionaceae and close to but not monophyletic with *Photobacterium* in the 44-taxon dataset as well as the frequent non-monophyly of *Photobacterium* based on the nested presence of *Aliivibrio*, and the non-monophyly of *Photobacterium* in [9] indicates that *Photobacterium*, as it is currently defined is not supported by phylogenetic evidence. A taxonomy of Vibrionaceae that reflects phylogeny is desirable and one of the conclusions of [9] was that more work must be done to clarify the relationships within *Photobacterium* because it was a paraphyletic assemblage in that analysis. By using genomic data here, it has become clearer that the differences among members of *Photobacterium* are stark and based on the data presented here, there is little evidence for its monophyly. Particularly since members of other genera, *S. costicola* and *G. hollisae*, are falling further to the base than members of *Photobacterium* and *Aliivibrio*, the validity of these other genera, *Salinivibrio* and *Grimontia*, whether they should be subsumed along with *Photobacterium* and *Aliivibrio* into *Vibrio*, or whether these should be maintained will require the further genome-scale analyses that include the remaining species of *Photobacterium*, *Salinivibrio*, and *Enterovibrio*.

Beyond the ability of genomes to provide improved taxonomy, the ability to integrate annotations with phylogenetic hypotheses across large numbers of species is the future of phylogenetic systematics. Here, by showing what is possible with multi–chromosomal bacterial genomes, that homologies can be made across genomes by not focusing on genes, that the topologies generated by these data are not found using collinear subsets of these data, but are found using random subsets of these data, future projects can be designed that will find the best species trees and avoid the problem of gene tree incongruence.

## Methods

### 19-taxon dataset

The 19-taxon dataset was separated into a large chromosome dataset, a small chromosome dataset, and a concatenated “both-chromosomes” dataset. In all cases, the entire *S. oneidensis* genome (a singe circular chromosome) was included as the outgroup. Primary homologies were calculated for each of the large and small chromosome datasets in Mauve [17]. Mauve is a genome alignment program that addresses the issue of genomic rearrangement by finding locally collinear blocks (LCBs), or contiguous segments of sequence within which there has not been rearrangement, but within a longer sequence that may have been subject to rearrangement events. The default parameters in Mauve were used as in [10]. Individual LCBs were then aligned with MAFFT v6.708-b [18]. Individual LCBs as well as concatenated datasets were subject to phylogenetic analysis using TNT (Maximum Parsimony; [19]) and Garli v2.0 multithreaded (Maximum Likelihood; [20]) or when alignments were longer than 500,000 bp, RaxML v7.2.8-alpha PTHREADS (Maximum Likelihood; [21]). For TNT, 1000 builds with SPR and TBR were followed by 1500 replications of ratchet and tree drifting [22]. Gaps were treated as a fifth state in TNT. For the 44-taxon datasets, additional TNT analyses were performed in which gaps were treated as missing. For Garli, the GTRGAMMA model was implemented and 20 replications were completed for each dataset. For RaxML, the GTRGAMMA model was implemented and 100 replications were completed for each dataset. LCB arrangement was plotted in circular view as in [10] in CGView [23].

As in [10], subset datasets were produced by randomly sampling nucleotides from concatenated LCB alignments for each chromosome using BioPerl scripts. These subset datasets were 10,000 bp, 20,000 bp, 30,000 bp 40,000 bp, 50,000 bp, 100,000 bp, 200,000 bp, 300,000 bp, 400,000 bp, 500,000 bp, and 1,000,000 bp (only up to 300,000 bp for the small chromosome because the concatenated alignment was only just over 400,000 bp). These datasets were each also analyzed in TNT and Garli or RaxML (depending on length).

### 44-taxon dataset

For this dataset, genomes were downloaded as detailed above or assembled *de novo* as detailed below. Because genome sequences that were present as multiple contigs were included, arrangement of these contigs was ignored and contigs were simply concatenated. Breakpoint analyses could not be completed on this dataset because the arrangement of gene and multi-gene fragments was not necessarily true to life after contig concatenation. A different strategy was implemented in Mauve in order to be able to include all 44 taxa. Concatenated contigs were grouped by two to three close relatives as determined in [9] as well the concatenated LCBs of closely related species from the Mauve results from the 19-taxon dataset. This was done because the *de novo* analysis in Mauve of all 44 concatenated genomes was computationally prohibitive. This strategy works because the Mauve results of interest are those LCBs common to all taxa. Since the 44-taxon dataset contains all the taxa of the 19-taxon dataset plus new taxa, one would expect the percent of base-pairs to be homologized by Mauve to decrease as taxa are added. By running Mauve analyses that start with the LCBs generated by the 19-taxon dataset Mauve analysis, one expects to capture the same homologies that one would capture if all 44-taxa were analyzed in Mauve from scratch. The LCBs that resulted from the smaller runs for all 44-taxa were extracted. Since Mauve provides results that collinearize the LCBs, a final, simpler Mauve run was performed with all 44 taxa together. The above was done separately for the large and small chromosomes. Phylogenetic analyses in TNT and Garli were conducted on the resulting alignments for both the large and small chromosomes.*V. brasiliensis* was removed from small chromosome dataset because it caused Mauve to crash repeatedly.

### New genome sequences

*Salinivibrio costicola* strain ATCC 33508, *Vibrio gazogenes* strain ATCC 43941, and *Aliivibrio logei* strain ATCC 35077 were ordered from the ATCC (American Type Culture Collection). They were grown on Difco Marine Agar. *S. costicola* was grown at 26 degrees C, *V. gazogenes* was grown at 26 degrees C and *A. logei* was grown at 18 degrees C. DNA was extracted using the Qiagen DNeasy DNA extraction kit and DNA concentration was measured using a Qubit 2.0 Fluorometer from Invitrogen. Qubit values were as follows: *S. costicola* = 266 ng/ml, *V. gazogenes* = 201 ng/ml, *A. logei* = 173 ng/ml. Paired-end 2*x*100 genome sequencing was performed with the Illumina HiSeq 2000 system at The University of Chicago Institute for Genomics and Systems Biology High-Throughput Genome Analysis Core. 139,917,975*x*2 100 bp sequences were generated for *S. costicola*, 88,859,684*x*2 were generated for *V. gazogenes*, 94,958,480*x*2 were generated for *A. logei*. The Geneious Assembler, part of Geneious v. 5.5 [24] was used to assemble the genomes on a Mac Pro with 8 dual-core processors and 96 GB RAM. The RAST annotation server was used to annotate assembled genomes [25].

## Competing interests

The authors declare that they have no competing interests.

## Authors’ contributions

RBD and WLS designed the study. RBD performed the analyses and wrote the manuscript. Both RBD and WLS have approved the final manuscript.

## Supplementary Material

Additional file 1 **Table S1.** Vibrionaceae 19–Taxon Large Chromosome Dataset LCBs and Trees. Click here for file

Additional file 2 **Table S2.** Vibrionaceae 19–Taxon Small Chromosome Dataset LCBs and Trees. Click here for file

Additional file 3 **Table S3.** Genes Found in RAST Subsystems for All Species Part 1. Click here for file

Additional file 4 **Table S6.** Vibrionaceae 19–Taxon Random Subset Datasets LCBs and Trees. Click here for file

Additional file 5 **Table S4.** Genes Found in RAST Subsystems for All Species Part 2. Click here for file

Additional file 6 **Table S5.** Genes Found in RAST Subsystems for All Species Part 3. Click here for file
