# Supplementary material for: Genome-level homology and phylogeny of Vibrionaceae (Gammaproteobacteria: Vibrionales) with three new complete genome sequences
Source: BMC Microbiol. 2013 Apr 11;13:80. doi: 10.1186/1471-2180-13-80 (PMC3663674; doi:10.1186/1471-2180-13-80)
Supplement: Additional file 1 — Table S1. Vibrionaceae 19–Taxon Large Chromosome Dataset LCBs and Trees. [file 1471-2180-13-80-S1.pdf]

Table S1: Vibrionaceae 19–taxon large chromosome dataset LCBs and trees.

LCB	Aligned bp	Algorithm	Tree topologies (Newick)
1	39,097	MP	(18,(17,((15,(13,14)),(16,((5,(4,((1,3),(2,0)))),((12,(10,11)),(6,(8,(7,9))))))))));
		ML	(18,(17,((16,(14,15)),(12,((4,(13,(0,(2,(1,3))))),((9,(10,11)),(5,(8,(6,7)))))))));
2	11,285	MP	(18,(17,((16,(14,15)),((11,(9,10)),(8,((7,(6,(4,5))),(13,(12,((1,3),(2,0))))))))));
		ML	(18,((17,(16,(14,15))),(12,(4,((13,(0,(2,(1,3)))),((10,(9,11)),(5,(6,(7,8)))))))));
3	13,886	MP	(18,(17,((16,(14,15)),((11,(9,10)),(8,((7,(4,(5,6))),(13,(12,((1,3),(2,0))))))))));
		ML	(18,(17,((12,(16,(14,15))),((11,(9,10)),(5,((7,(6,8)),(4,(13,(0,(2,(1,3)))))))))));
4	1,030	MP	(18,(17,((16,(14,15)),((11,(9,10)),(8,((7,(4,(5,6))),(13,(12,((1,3),(2,0))))))))));
		ML	(18,(3,2,1,(0,(13,(4,((11,10,9),((5,(6,(7,8))),(12,(17,(16,(14,15))))))))))));
5	4,129	MP	(18,((17,(16,(14,15))),(13,(12,(((2,3),(1,0)),(4,((11,(9,10)),(8,(6,(5,7))))))))));
		ML	(18,(12,(((9,(10,11)),(5,(6,(7,8)))),((4,(17,(16,(14,15)))),(13,(0,(3,2,1)))))));
6	3,801	MP	(18,((14,(12,13)),(16,((15,(4,(0,(3,(1,2))))),(17,((11,(9,10)),(5,(8,(6,7)))))))));
		MP	(18,((14,(12,13)),(16,((15,(4,(0,(3,(1,2))))),(17,((9,(10,11)),(5,(8,(6,7)))))))));
		ML	(18,((16,(14,15)),(17,(12,((8,(5,(6,7))),((9,(10,11)),(4,(13,(1,0,3,2)))))))));
7	7,936	MP	(18,((0,(1,(2,3))),(4,(13,((12,(17,(16,(14,15)))),((10,(9,11)),(8,(6,(5,7)))))))));
		MP	(18,((17,(16,(14,15))),(13,((0,1,2,3),(4,(12,((11,(9,10)),(8,(5,(6,7))))))))));
		ML	(18,((0,(2,(1,3))),(4,(13,((10,(9,11)),((8,(5,(6,7))),(12,(17,(16,(14,15))))))))));
8	12,328	MP	(18,((16,(14,15)),(17,(13,((5,(4,(2,(1,(3,0))))),((10,(11,12)),(9,(8,(6,7)))))))));
		ML	(18,((9,(10,11)),((6,(5,(7,8))),(12,((17,(16,(14,15))),(4,(13,(0,3,1,2))))))));
9	7,326	MP	(18,((16,(14,15)),(17,(13,((5,(4,(2,(1,(3,0))))),((10,(11,12)),(9,(6,(7,8)))))))));
		ML	(18,((17,(16,(14,15))),((4,(13,(0,2,3,1))),(12,((10,(9,11)),(5,(6,(7,8))))))));
10	40,528	MP	(18,(17,((16,(14,15)),(((12,13),((2,3),(1,0))),(11,(7,((4,(5,6)),(9,(8,10)))))))));
		ML	(18,((17,(16,(14,15))),((4,(13,(0,(2,(1,3))))),((9,(10,11)),(12,(8,(6,(5,7))))))));
11	7,444	MP	(18,((17,(16,(14,15))),((2,(3,(1,0))),(12,(13,(11,(7,((4,(5,6)),(9,(8,10))))))))));
		MP	(18,(17,(((16,(14,15)),(11,(13,(6,(5,7))))),((4,(10,(8,9))),(12,(2,(1,(3,0))))))));
		ML	(18,(17,((16,(14,15)),(12,((4,(13,(0,(3,1,2)))),((11,(9,10)),((7,8),(5,6))))))));
12	1,325	MP	(18,(17,(((16,(14,15)),(11,(13,(7,(5,6))))),((4,(10,(8,9))),(12,(2,(1,(3,0))))))));
		ML	(18,(17,((16,(14,15)),(12,((5,(7,(6,8))),((10,(9,11)),(4,(13,(2,3,0,1)))))))));
13	6,619	MP	(18,(17,((16,(14,15)),(11,(13,((12,(3,(2,(1,0)))),((10,(8,9)),(7,(4,(5,6))))))))));
		ML	(18,(17,((16,(14,15)),(12,((4,(13,(0,2,(1,3)))),((10,(9,11)),(5,(7,(6,8)))))))));
14	6,341	MP	(18,(17,((16,(14,15)),(11,(13,((12,(3,(2,(1,0)))),((10,(8,9)),(4,(6,(5,7))))))))));
		ML	(18,((17,(16,(14,15))),(12,((4,(13,(3,1,(0,2)))),((11,(9,10)),(5,(7,(6,8))))))));
15	4,806	MP	(18,((16,(14,15)),(17,(((8,(9,10)),(7,(4,(5,6)))),(11,(12,(13,(0,(3,(1,2))))))))));
		ML	(18,((17,(16,(14,15))),((4,(13,(2,3,0,1))),(12,((10,(9,11)),(5,(8,(6,7))))))));
16	4,942	MP	(18,((16,(14,15)),(17,(((8,(9,10)),(4,(7,(5,6)))),(11,(12,(13,(0,(3,(1,2))))))))));
		ML	(18,((17,(16,(14,15))),(12,((4,(13,(1,3,(0,2)))),((9,(10,11)),(5,(8,(6,7))))))));
17	7,302	MP	(18,(6,((1,(3,(2,0))),(12,(17,((4,16),((5,(7,8)),((10,(9,11)),(15,(13,14))))))))));
		ML	(18,(17,((16,(14,15)),(12,((4,(13,(1,0,2,3))),((11,(9,10)),(5,(8,(6,7)))))))));
18	4,390	MP	(18,(6,((1,(3,(2,0))),(12,(17,((4,16),((5,(7,8)),((11,(9,10)),(15,(13,14))))))))));

Table S1: Vibrionaceae 19–taxon large chromosome dataset LCBs and trees (continued).

LCB	Aligned bp	Algorithm	Tree topologies (Newick)
		ML	(18,(13,((4,(0,2,1,3)),(12,((17,(16,(14,15))),((10,(9,11)),(5,(8,(6,7)))))))));
19	15,459	MP	(18,((14,(17,(15,16))),(13,((12,(4,((2,3),(1,0)))),((11,(9,10)),(5,(7,(6,8))))))));
		ML	(18,((17,(16,(14,15))),(12,((4,(13,(0,(2,1,3)))),((11,(9,10)),(5,(8,(6,7))))))));
20	12,293	MP	(18,(5,(10,((17,(16,(14,15))),((4,(0,(1,(2,3)))),((12,(11,13)),(6,(7,(8,9)))))))));
		ML	(18,(17,((13,(4,(0,2,3,1))),((11,(9,10)),((8,(6,7)),(5,(12,(16,(14,15)))))))));
21	19,929	MP	(18,(5,(10,((17,(16,(14,15))),((4,(0,(1,(2,3)))),((11,(12,13)),(6,(7,(8,9)))))))));
		ML	(18,((13,(0,(3,2,1))),(4,((17,(16,(14,15))),((11,(9,10)),(12,(8,(5,(6,7)))))))));
22	33,541	MP	(18,(13,((17,(16,(14,15))),(4,(9,((3,(0,(1,2))),((12,(10,11)),(8,(7,(5,6)))))))));
		ML	(18,(4,(13,(0,(2,3,1))),((12,(17,(16,(14,15)))),((9,(10,11)),(8,(5,(6,7)))))));
23	151,773	MP	(18,(13,((17,(16,(14,15))),(4,((3,(0,(1,2))),(9,((12,(10,11)),(8,(7,(5,6))))))))));
		ML	(18,((17,(16,(14,15))),(12,((5,(6,(7,8))),((10,(9,11)),(4,(13,(0,(2,3,1)))))))));
24	5,189	MP	(18,(17,((16,(14,15)),(13,((5,(4,((2,3),(1,0)))),((10,(11,12)),(9,(6,(7,8)))))))));
		MP	(18,(17,((16,(14,15)),(13,((5,(4,((2,3),(1,0)))),((12,(10,11)),(9,(6,(7,8)))))))));
		ML	(18,(17,((16,(14,15)),((9,(10,11)),((12,(5,(8,(6,7)))),(4,(13,(0,(1,2,3)))))))));
25	6,526	MP	(18,(15,(14,(16,(17,((13,(12,(0,(2,(1,3))))),((7,(4,(5,6))),(11,(8,(9,10))))))))));
		ML	(18,(17,((16,(14,15)),(12,((4,(13,(0,(3,1,2)))),((10,(9,11)),(5,(8,(6,7)))))))));
26	42,063	MP	(18,(15,(14,(16,(17,((13,(12,(0,(2,(1,3))))),((7,(4,(5,6))),(11,(10,(8,9))))))))));
		MP	(18,(17,((16,(14,15)),((4,(0,(1,(2,3)))),(13,((11,(9,10)),(12,(8,(5,(6,7))))))))));
		ML	(18,((17,(16,(14,15))),((4,(13,(0,(2,(1,3))))),(12,((10,(9,11)),(5,(8,(6,7))))))));
27	11,968	MP	(18,((17,(16,(14,15))),((4,(0,(1,(2,3)))),(13,((11,(9,10)),(12,(8,(5,(6,7)))))))));
		ML	(18,(17,((16,(14,15)),(12,((4,(13,(0,(1,2,3)))),((9,(10,11)),(8,(7,(5,6)))))))));
28	15,438	MP	(18,(5,((17,(16,(14,15))),((4,(1,(0,(2,3)))),(13,((12,(10,11)),(9,(6,(7,8)))))))));
		ML	(18,((16,(14,15)),(17,(12,((5,(8,(6,7))),((9,(10,11)),(4,(13,(0,(2,3,1))))))))));
29	6,474	MP	(18,(5,((17,(16,(14,15))),((4,(2,(3,(1,0)))),(13,((12,(10,11)),(9,(6,(7,8)))))))));
		ML	(18,(17,((16,(14,15)),((4,(13,(0,(3,2,1)))),((9,(10,11)),(12,(8,(5,(6,7)))))))));
30	9,101	MP	(18,(5,((17,(16,(14,15))),((4,((2,3),(1,0))),(13,((12,(10,11)),(9,(6,(7,8)))))))));
		ML	(18,(17,((16,(14,15)),((4,(13,(0,(3,2,1)))),(12,((10,(9,11)),(5,(8,(6,7)))))))));
31	3,546	MP	(18,(17,((15,(13,14)),((5,(4,(3,(1,(2,0))))),(6,((12,(10,11)),(16,(9,(7,8)))))))));
		MP	(18,(17,((15,(13,14)),((4,(5,(3,(1,(2,0))))),(6,((12,(10,11)),(16,(9,(7,8)))))))));
		ML	(18,((16,(14,15)),(17,((4,(13,(0,(3,2,1)))),(12,((9,(10,11)),(5,(8,(6,7)))))))));
32	6,854	MP	(18,(17,((16,(14,15)),(12,((13,(4,(2,(1,(3,0))))),((9,(10,11)),(5,(8,(6,7)))))))));
		ML	(18,(17,((16,(14,15)),(12,((4,(13,(0,(3,2,1)))),((11,(9,10)),(5,(6,(7,8)))))))));
33	2,118	MP	(18,(17,((16,(14,15)),((13,(4,(2,(1,(3,0))))),(12,((11,(9,10)),(5,(8,(6,7)))))))));
		ML	(18,((5,(8,(6,7))),((12,(17,(16,(14,15)))),((11,(9,10)),(13,(4,(1,0,3,2)))))));
34	1,271	MP	(18,(17,((16,(14,15)),((13,(4,(2,(1,(3,0))))),(12,((9,(10,11)),(5,(8,(6,7)))))))));
		ML	(18,(12,((17,(16,(14,15))),((4,(13,(0,(1,3,2)))),((11,(9,10)),(5,(6,(7,8))))))));
35	8,075	MP	(18,((14,(17,(15,16))),(13,((12,(4,((2,3),(1,0)))),((10,(9,11)),(5,(7,(6,8))))))));
		ML	(18,((16,(14,15)),(17,(12,((5,(6,(7,8))),((10,(9,11)),(4,(13,(0,(2,(1,3)))))))))));
36	13,650	MP	(18,((14,(17,(15,16))),(13,((12,(4,((2,3),(1,0)))),((9,(10,11)),(5,(7,(6,8))))))));

Table S1: Vibrionaceae 19–taxon large chromosome dataset LCBs and trees (continued).

LCB	Aligned bp	Algorithm	Tree topologies (Newick)
		ML	(18,(17,((16,(14,15)),((4,(13,(0,(1,3,2)))),(12,((10,(9,11)),((6,7),(5,8))))))));
37	4,652	MP	(18,((17,(15,16)),(((14,(12,13)),(7,(8,(9,10)))),((6,11),(5,(4,((1,3),(2,0))))))));
		ML	(18,((10,(9,11)),((13,(0,(2,3,1))),((5,(8,(6,7))),(4,(12,(17,(16,(14,15)))))))));
38	8,966	MP	(18,((17,(15,16)),(((14,(12,13)),(7,(10,(8,9)))),((6,11),(5,(4,((1,3),(2,0))))))));
		ML	(18,((17,(16,(14,15))),((10,(9,11)),(12,(((7,8),(5,6)),(4,(13,(0,(2,1,3)))))))));
39	6,294	MP	(18,(17,((15,(13,14)),(16,((5,(4,((1,3),(2,0)))),((12,(10,11)),(6,(7,(8,9)))))))));
		ML	(18,((4,(13,(0,(3,1,2)))),((10,(9,11)),((8,(7,(5,6))),(12,(17,(16,(14,15))))))));
40	13,163	MP	(18,(14,(13,(15,(16,(17,(11,(12,((0,(3,(1,2))),((9,(8,10)),(4,(6,(5,7))))))))))));
		ML	(18,(17,((16,(14,15)),(12,((4,(13,(2,(0,(1,3))))),((9,(10,11)),(5,(6,(7,8)))))))));
41	35,664	MP	(18,(14,(13,(15,(16,(17,(11,(12,((0,(3,(1,2))),((8,(9,10)),(4,(6,(5,7))))))))))));
		ML	(18,((2,(0,(1,3))),(4,(13,((17,(16,(14,15))),(12,((11,(9,10)),(5,(6,(7,8))))))))));
42	3,886	MP	(18,((16,17),(15,((4,(5,13)),((14,((2,3),(1,0))),((12,(10,11)),(9,(6,(7,8)))))))));
		ML	(18,((10,(9,11)),(13,(4,((0,2,3,1),(12,(5,((8,(6,7)),(17,(16,(14,15))))))))));
43	5,522	MP	(18,((16,17),(15,((4,(5,13)),((14,(3,(1,(2,0)))),((12,(10,11)),(9,(6,(7,8)))))))));
		ML	(18,(17,((16,(14,15)),(12,(4,((13,(3,2,0,1)),((10,(9,11)),(5,(7,(6,8))))))))));
44	6,663	MP	(18,((16,(14,15)),((6,17),((0,(2,(1,3))),((9,(5,(7,8))),(13,(4,(12,(10,11)))))))));
		ML	(18,((17,(16,(14,15))),((12,(5,(8,(6,7)))),((11,(9,10)),(4,(13,(1,0,3,2)))))));
45	9,340	MP	(18,((16,(14,15)),((6,17),((0,(2,(1,3))),((9,(5,(7,8))),(13,(4,(11,(10,12)))))))));
		ML	(18,((4,(13,(0,(2,(1,3))))),((11,(9,10)),((8,(6,(5,7))),(17,(12,(16,(14,15))))))));
46	27,388	MP	(18,((13,(15,16)),(((11,(9,10)),(4,(6,(5,7)))),(14,(17,(12,(8,((1,3),(2,0)))))))));
		ML	(18,((17,(16,(14,15))),(12,(4,((13,(10,(9,11))),((6,(5,(7,8))),(0,(3,1,2))))))));
47	8,922	MP	(18,((13,(15,16)),(((11,(9,10)),(7,(4,(5,6)))),(14,(17,(12,(8,((1,3),(2,0)))))))));
		ML	(18,(17,(12,((16,(14,15)),((5,(7,(6,8))),((9,(10,11)),(4,(13,(3,0,1,2)))))))));
48	11,129	MP	(18,((17,(16,(14,15))),((10,(9,(7,8))),((11,(12,13)),(6,(5,(4,(2,(3,(1,0))))))))));
		ML	(18,((17,(16,(14,15))),(12,((5,(7,(6,8))),((10,(9,11)),(4,(13,((1,3),(0,2)))))))));
49	18,293	MP	(18,((17,(16,(14,15))),((10,(9,(7,8))),((13,(11,12)),(6,(5,(4,(2,(3,(1,0))))))))));
		ML	(18,((17,(16,(14,15))),((4,(13,((1,3),(0,2)))),(12,((10,(9,11)),((5,8),(6,7)))))));
50	7,656	MP	(18,((17,(10,(8,(4,5)))),((16,(14,15)),(((1,2),(3,0)),(13,((6,7),(9,(11,12))))))));
		ML	(18,(17,((16,(14,15)),((4,(13,(0,(1,2,3)))),(12,((11,(9,10)),(5,(8,(6,7)))))))));
51	18,272	MP	(18,((17,(10,(5,(4,8)))),((16,(14,15)),(((1,2),(3,0)),(13,((6,7),(9,(11,12))))))));
		ML	(18,((17,(16,(14,15))),(12,((4,(13,(0,(1,3,2)))),((10,(9,11)),(5,(8,(6,7))))))));
52	23,997	MP	(18,((17,(16,(14,15))),((0,(1,(2,3))),(4,(5,(13,((12,(10,11)),(6,(9,(7,8))))))))));
		ML	(18,(17,((16,(14,15)),(4,(12,((5,(8,(6,7))),((9,(10,11)),(13,(0,3,1,2)))))))));
53	8,188	MP	(18,((17,(16,(14,15))),((0,(1,(2,3))),(4,(5,(13,((11,(10,12)),(6,(9,(7,8))))))))));
		ML	(18,((4,(12,(17,(16,(14,15))))),((13,((1,3),(0,2))),((9,(10,11)),(8,(5,(6,7)))))));
54	27,651	MP	(18,((16,17),((6,(9,(7,8))),(13,(((10,15),(14,(11,12))),(5,(4,((2,3),(1,0)))))))));
		ML	(18,((17,(16,(14,15))),(12,((4,(13,(0,(2,(1,3))))),((10,(9,11)),(5,(7,(6,8))))))));
55	8,887	MP	(18,((16,17),((6,(9,(7,8))),(13,(((10,15),(14,(11,12))),(5,(4,(2,(3,(1,0))))))))));
		ML	(18,(1,3,((0,2),(13,((4,(12,(17,(16,(14,15))))),((10,(9,11)),(8,(7,(5,6)))))))));

Table S1: Vibrionaceae 19–taxon large chromosome dataset LCBs and trees (continued).

LCB	Aligned bp	Algorithm	Tree topologies (Newick)
56	18,231	MP	(18,((16,17),((6,(9,(7,8))),(13,(((10,15),(14,(11,12))),(5,(4,(0,(1,(2,3))))))))));
		ML	(18,((17,(16,(14,15))),(((9,(10,11)),(5,(8,(6,7)))),(12,(4,(13,((1,3),(0,2)))))))));
57	9,123	MP	(18,((17,(16,(14,15))),((13,(4,((2,3),(1,0)))),(12,((9,(10,11)),(5,(6,(7,8)))))))));
		ML	(18,((11,(9,10)),((4,(13,(0,(2,(1,3))))),((5,(6,(7,8))),(12,(17,(16,(14,15))))))));
58	2,751	MP	(18,((17,(16,(14,15))),((13,(4,(2,(3,(1,0))))),(12,((9,(10,11)),(5,(6,(7,8)))))))));
		ML	(18,((13,(0,(1,3,2))),((17,(16,(14,15))),(12,(5,((6,7),(8,(4,(10,(9,11))))))))));
59	2,222	MP	(18,((17,(16,(14,15))),((13,(4,(0,(1,(2,3))))),(12,((9,(10,11)),(5,(6,(7,8)))))))));
		ML	(18,((0,(3,1,2)),((13,(17,(16,(14,15)))),((4,(11,(9,10))),(12,(7,(5,(6,8))))))));
60	4,774	MP	(18,(17,((5,(4,(3,(2,(1,0))))),((16,(14,15)),(10,((13,(11,12)),(8,(7,(6,9)))))))));
		MP	(18,(17,((5,(4,(3,(2,(1,0))))),((16,(14,15)),(10,((11,(12,13)),(8,(7,(6,9)))))))));
		ML	(18,(((10,(9,11)),(5,(7,(6,8)))),((17,(16,(14,15))),(12,(4,(13,(3,(2,0,1))))))));
61	2,157	MP	(18,(17,((16,(14,15)),(13,((5,(4,(2,(1,(3,0))))),((12,(10,11)),(6,(9,(7,8)))))))));
		ML	(18,((16,(14,15)),(17,(12,((4,(13,(0,1,2,3))),((9,(10,11)),(5,(6,(7,8)))))))));
62	9,904	MP	(18,(17,((16,(14,15)),(13,((5,(4,(0,(3,(1,2))))),((12,(10,11)),(6,(9,(7,8)))))))));
		ML	(18,(17,((16,(14,15)),((4,(13,(0,(3,2,1)))),(12,((10,(9,11)),(5,(8,(6,7)))))))));
63	2,266	MP	(18,((16,(14,15)),(17,((5,(4,(0,(1,(2,3))))),(13,((11,(10,12)),(6,(9,(7,8)))))))));
		ML	(18,((0,(2,1,3)),(13,((4,(12,(17,(16,(14,15))))),((9,(10,11)),(7,(8,(5,6))))))));
64	2,722	MP	(18,((16,(14,15)),(17,((5,(4,(0,(1,(2,3))))),(13,((12,(10,11)),(6,(9,(7,8)))))))));
		ML	(18,(13,((0,(3,2,1)),(4,((17,(16,(14,15))),((10,(9,11)),(12,(8,(7,(5,6))))))))));
65	1,014	MP	(18,((17,(16,(14,15))),(13,((5,(4,(0,(3,(1,2))))),((10,(11,12)),(6,(9,(7,8))))))));
		ML	(18,((13,(0,(2,3,1))),(4,((12,(17,(16,(14,15)))),((10,(9,11)),(5,8,(6,7)))))));
66	12,082	MP	(18,(14,(17,((15,16),(5,(13,(9,(6,(8,((7,(10,(11,12))),(4,(0,(2,(1,3))))))))))))));
		ML	(18,((17,(16,(14,15))),((13,(4,(0,(2,1,3)))),(12,((11,(9,10)),(5,(6,(7,8)))))))));
67	8,773	MP	(18,((16,17),(4,(15,(14,(13,((7,(5,6)),(8,(10,((9,11),(12,(0,(1,(2,3))))))))))))));
		ML	(18,((17,(16,(14,15))),(12,((4,(13,(3,0,2,1))),((10,(9,11)),(5,(8,(6,7)))))))));
68	5,049	MP	(18,(14,((16,(15,17)),(7,(8,((5,6),((10,(11,12)),(9,(13,(4,(3,(0,(1,2)))))))))))));
		ML	(18,((17,(16,(14,15))),((8,(5,(6,7))),(12,((11,(9,10)),(4,(13,(0,(3,1,2))))))))));
69	1,146	MP	(18,((10,(11,12)),(4,(9,(8,(7,(6,(13,((17,(16,(14,15))),(5,(3,(0,(1,2)))))))))))));
		ML	(18,((11,(9,10)),((17,(16,(14,15))),((8,(12,(6,(5,7)))),(4,(13,(3,1,0,2)))))));
70	19,144	MP	(18,(13,(12,(8,(6,((9,(10,11)),(7,((4,5),((16,(14,15)),(17,(0,(3,(1,2)))))))))))));
		ML	(18,((13,(0,(3,2,1))),(4,((10,(9,11)),((5,(8,(6,7))),(12,(17,(16,(14,15)))))))));
71	11,033	MP	(18,((17,(15,16)),(14,(8,((6,7),(9,((12,(10,11)),(13,(4,(5,(2,(3,(1,0)))))))))))));
		ML	(18,((17,(16,(14,15))),((4,(13,(0,(2,(1,3))))),(12,((9,(10,11)),(5,(8,(6,7)))))))));
72	7,215	MP	(18,((17,(16,(14,15))),((9,(10,11)),(12,(5,(7,(6,(8,(13,(4,(0,(3,(1,2))))))))))))));
		ML	(18,((17,(16,(14,15))),(12,((4,(13,(0,(3,1,2)))),((10,(9,11)),(5,(8,(6,7)))))))));
73	5,633	MP	(18,((7,(5,6)),(10,(11,(12,(9,(13,(4,(8,((17,(16,(14,15))),(0,(3,(1,2)))))))))))));
		ML	(18,((16,(14,15)),(17,((4,(13,(2,0,1,3))),(12,((9,(10,11)),(8,(7,(5,6)))))))));
74	6,206	MP	(18,(17,(8,(7,(6,(5,((13,(14,15)),((12,(11,(9,10))),(16,(4,(0,(2,(1,3)))))))))))));
		MP	(18,((16,(14,15)),(8,(9,((0,(3,(1,2))),(13,(6,(5,(7,(4,(17,(12,(10,11)))))))))))));

Table S1: Vibrionaceae 19–taxon large chromosome dataset LCBs and trees (continued).

LCB	Aligned bp	Algorithm	Tree topologies (Newick)
		ML	(18,((1,0,2,3),(13,(4,((10,(9,11)),(7,(8,(6,(5,(12,(17,(16,(14,15))))))))))));
75	4,986	MP	(18,((0,(2,(1,3))),(5,((16,(14,15)),(4,(13,(17,(9,(7,(6,(8,(12,(10,11)))))))))))));
		ML	(18,(6,(5,7),(8,((10,(9,11)),((12,(17,(16,(14,15)))),(4,(13,(0,(1,2,3)))))))));
76	6,270	MP	(18,(13,((8,(9,10)),(6,(4,(5,(7,(11,(12,((17,(16,(14,15))),(1,(0,(2,3)))))))))))));
		ML	(18,((16,(14,15)),(17,(12,((4,(13,(0,(2,(1,3))))),((11,(9,10)),(8,(5,(6,7)))))))));
77	5,818	MP	(18,(13,((2,(1,(3,0))),(4,((11,(9,10)),((7,8),(6,(5,(12,(14,(17,(15,16))))))))))));
		ML	(18,(17,(12,(((11,(9,10)),(8,(7,(5,6)))),((4,(16,(14,15))),(13,(3,0,1,2)))))));
78	2,713	MP	(18,((16,17),(15,(14,((5,(6,(7,8))),((11,(9,10)),(12,(13,(4,(3,(2,(1,0))))))))))));
		ML	(18,(13,((3,(1,0,2)),(4,((12,(17,(16,(14,15)))),((11,(9,10)),(5,(6,(7,8)))))))));
79	7,416	MP	(18,((17,(16,(14,15))),(12,((10,(8,9)),(4,(7,((5,6),(11,(13,(0,(3,(1,2)))))))))))));
		ML	(18,((4,(1,0,3,2)),(13,((12,(17,(16,(14,15)))),((9,(10,11)),(7,(8,(5,6))))))));
80	1,908	MP	(18,((16,17),(13,(10,(15,((8,9),(12,((6,(5,7)),((4,(11,14)),(3,(2,(1,0)))))))))))));
		ML	(18,((17,(16,(14,15))),((4,(13,(0,1,2,3))),(12,((10,(9,11)),(5,(8,(6,7))))))));
81	6,785	MP	(18,((17,(16,(14,15))),(13,((10,(11,12)),((7,8),(6,(9,(5,(4,(0,(3,(1,2)))))))))))));
		ML	(18,(12,(((10,(9,11)),(5,(7,(6,8)))),((17,(16,(14,15))),(4,(13,(0,1,3,2)))))));
82	11,551	MP	(18,(4,(9,((1,(2,(3,0))),(8,(5,((6,7),((12,(10,11)),(17,(16,(15,(13,14)))))))))))));
		ML	(18,(17,((16,(14,15)),(12,((4,(13,(0,2,(1,3)))),((10,(9,11)),((5,6),(7,8))))))));
83	3,374	MP	(18,(4,((12,(17,(16,(14,15)))),(9,(11,(10,(8,((7,(5,6)),(13,(1,(0,(2,3)))))))))))));
		ML	(18,((17,(16,(14,15))),(12,((4,(13,(2,0,3,1))),((9,(10,11)),(5,(8,(6,7))))))));
84	4,934	MP	(18,(((1,3),(2,0)),((17,(14,(12,13))),(11,(7,(15,(16,(6,(4,(5,(10,(8,9)))))))))))));
		ML	(18,(17,((16,(14,15)),((4,(13,(0,3,2,1))),(12,((9,(10,11)),(5,(8,(6,7)))))))));
85	3,660	MP	(18,(17,((16,(14,15)),(6,((7,(8,9)),((11,(10,12)),(13,(5,(4,(0,(3,(1,2)))))))))))));
		ML	(18,(3,1,0,2,(4,(13,((12,(17,(16,(14,15)))),((10,(9,11)),(5,(7,(6,8)))))))));
86	4,925	MP	(18,((14,15),(16,(17,(10,(5,((0,(1,(2,3))),(4,((11,(12,13)),(6,(9,(7,8)))))))))))));
		ML	(18,(5,((8,(6,7)),((11,9,10),(12,(4,((17,(16,(14,15))),(13,(3,1,0,2)))))))));
87	2,649	MP	(18,(16,(17,((14,15),(13,((6,(5,(7,8))),((11,(9,10)),(12,(4,(0,(2,(1,3)))))))))))));
		ML	(18,(4,((1,0,2,3),((13,(8,(10,(9,11)))),((17,(16,(14,15))),(12,(5,(6,7))))))));
88	11,448	MP	(18,((14,15),(11,((0,(1,(2,3))),(5,(12,(6,(7,((10,(8,9)),(17,(13,(4,16)))))))))))));
		ML	(18,(((11,(9,10)),(5,(7,(6,8)))),((12,(17,(16,(14,15)))),(4,(13,(0,(3,2,1)))))));
89	1,095	MP	(18,((4,(2,(1,(3,0)))),(17,(13,((16,(14,15)),(12,(8,(7,(6,(5,(9,(10,11))))))))))));
		ML	(18,(17,((16,(14,15)),((13,(1,0,2,3)),((4,12),((11,(9,10)),(5,(7,(6,8)))))))));
90	36,613	MP	(18,((17,(16,(14,15))),(9,(8,(7,((5,6),((12,(10,11)),(13,(4,(1,(3,(2,0))))))))))));
		ML	(18,(17,((6,(8,(5,7))),((12,(16,(14,15))),((11,(9,10)),((4,13),(0,2,3,1)))))));
91	68,050	MP	(18,(17,(12,((11,(9,10)),(5,((6,(7,8)),((16,(14,15)),(13,(4,(3,(0,(1,2)))))))))))));
		ML	(18,(17,((16,(14,15)),((4,(13,(2,0,3,1))),(12,((11,(9,10)),((7,8),(5,6))))))));
92	13,435	MP	(18,((16,17),((12,(10,11)),(14,(13,((9,(6,(7,8))),(15,(5,(4,(0,(1,(2,3)))))))))))));
		ML	(18,((17,(16,(14,15))),((12,(5,(6,(7,8)))),((9,(10,11)),(4,(13,(0,(2,(1,3)))))))));
93	15,616	MP	(18,((16,(14,15)),(17,(13,(9,((6,(7,8)),((12,(10,11)),(4,(5,(0,(2,(1,3)))))))))))));
		ML	(18,((0,3,1,2),(4,(13,((17,(16,(14,15))),(12,((11,(9,10)),(8,(6,(5,7))))))))));

Table S1: Vibrionaceae 19–taxon large chromosome dataset LCBs and trees (continued).

LCB	Aligned bp	Algorithm	Tree topologies (Newick)
94	18,040	MP	(18,(15,(14,(16,(17,((4,((2,3),(1,0))),((13,(11,12)),(10,(8,(6,(5,(7,9)))))))))));
		ML	(18,((17,(16,(14,15))),(12,((4,(13,(0,(1,2,3)))),((10,(9,11)),(8,(5,(6,7))))))));
95	6,918	MP	(18,(15,((14,(12,13)),((10,(8,9)),(7,((11,17),(4,((5,6),(16,(0,(1,(2,3)))))))))));
		ML	(18,(17,((16,(14,15)),((5,(8,(6,7))),(12,((10,(9,11)),(4,(13,(2,0,3,1)))))))));
96	4,559	MP	(18,((16,17),(14,(15,(13,((5,(4,(2,(1,(3,0))))),((12,(10,11)),(6,(9,(7,8)))))))));
		ML	(18,(5,((7,(6,8)),((4,(13,(0,(1,2,3)))),(12,((11,(9,10)),(17,(16,(14,15)))))))));
97	8,226	MP	(18,(17,((16,(14,15)),(12,((11,(8,(9,10))),((7,(5,6)),(13,(4,(0,(1,(2,3))))))))));
		ML	(18,(17,((16,(14,15)),((4,12),((13,(0,1,2,3)),((11,(9,10)),(5,(6,(7,8)))))))));
98	52,599	MP	(18,(17,((16,(14,15)),(12,((11,(9,10)),((0,(2,(1,3))),(4,(13,(5,(8,(6,7))))))))));
		ML	(18,((1,0,2,3),(13,(4,((9,(10,11)),((6,(7,(5,8))),(12,(17,(16,(14,15))))))))));
99	10,475	MP	(18,(17,((2,(1,(3,0))),(8,(12,(13,(6,((4,(5,7)),((11,(9,10)),(16,(14,15)))))))))));
		ML	(18,(17,((16,(14,15)),(12,((4,(13,(0,(1,3,2)))),((9,(10,11)),(5,(8,(6,7)))))))));
100	3,918	MP	(18,(17,((16,(14,15)),((12,(10,11)),((9,(8,(6,7))),(13,(5,(4,(0,(3,(1,2)))))))))));
		ML	(18,(5,(6,(7,8)),((10,(9,11)),((12,(17,(16,(14,15)))),((4,13),(0,(2,3,1)))))));
101	18,186	MP	(18,(17,((16,(14,15)),(9,(5,((8,(6,7)),((12,(10,11)),(13,(4,((2,3),(1,0)))))))))));
		ML	(18,((17,(16,(14,15))),((4,(13,(1,0,2,3))),(12,((11,(9,10)),(5,(7,(6,8))))))));
102	4,439	MP	(18,(17,((16,(14,15)),(13,(9,((0,(3,(1,2))),(4,((11,(10,12)),(8,(7,(5,6)))))))))));
		ML	(18,(16,((14,15),(12,((5,(8,(6,7))),((10,(9,11)),((4,17),(13,(0,(2,3,1))))))))));
103	4,574	MP	(18,(16,((17,(14,15)),(13,((5,(6,(7,8))),((9,(10,11)),(12,(4,(3,(1,(2,0)))))))))));
		MP	(18,((14,15),(16,(17,(((10,(8,9)),(4,(7,(5,6)))),(11,(12,(13,(1,(3,(2,0)))))))))));
		ML	(18,(13,(0,(2,3,1)),(4,((12,(17,(16,(14,15)))),((11,(9,10)),(5,(7,(6,8))))))));
104	9,325	MP	(18,((16,17),(15,((8,(6,7)),((11,(12,(9,10))),((5,13),(14,(4,(0,(3,(1,2)))))))))));
		ML	(18,(15,(14,((16,17),((4,(13,(3,(0,1,2)))),((10,(9,11)),(5,(6,(7,(8,12))))))))));
105	37,144	MP	(18,((15,(13,14)),(9,(17,(16,(4,((5,(3,(0,(1,2)))),((6,(7,8)),(12,(10,11))))))))));
		ML	(18,((0,(1,3,2)),(4,(13,((17,(16,(14,15))),(12,((10,(9,11)),(5,(8,(6,7))))))))));
106	3,855	MP	(18,(17,((16,(14,15)),((9,(6,(7,8))),((11,(10,12)),(13,(5,(4,(1,(0,(2,3)))))))))));
		ML	(18,(12,((17,(16,(14,15))),(5,((6,8),(7,((11,(9,10)),(4,(13,(0,2,1,3))))))))));
107	19,284	MP	(18,(16,(8,((7,(5,6)),((12,(17,(14,15))),(13,((11,(9,10)),(4,(3,(1,(2,0)))))))))));
		ML	(18,((13,(4,(0,(3,1,2)))),((12,(17,(16,(14,15)))),((10,(9,11)),(5,(8,(6,7)))))));
108	19,382	MP	(18,(17,((11,(9,10)),(5,(13,(4,((3,(0,(1,2))),(12,((8,(6,7)),(16,(14,15)))))))))));
		ML	(18,((4,(13,(0,(2,3,1)))),((9,(10,11)),(17,16,(14,15)),(12,(5,(8,(6,7)))))));
109	27,162	MP	(18,((16,17),(14,(15,(13,((5,(4,(1,(3,(2,0))))),((12,(10,11)),((6,7),(8,9)))))))));
		ML	(18,((17,(16,(14,15))),(12,((8,(6,(5,7))),((10,(9,11)),(4,(13,(0,(3,1,2)))))))));
110	8,639	MP	(18,(11,((14,(12,13)),((9,(6,(7,8))),(10,((17,(15,16)),(5,(4,(1,(2,(3,0)))))))))));
		ML	(18,(17,((16,(14,15)),(((6,(5,7)),(11,(9,10))),((8,12),(4,(13,(0,2,1,3))))))));
111	37,180	MP	(18,(14,((17,(15,16)),(13,(5,(4,((1,(3,(2,0))),((8,(6,7)),(12,(11,(9,10)))))))))));
		ML	(18,((17,(16,(14,15))),(4,((13,((1,3),(0,2))),(12,((10,(9,11)),(5,(6,(7,8)))))))));
112	8,583	MP	(18,(17,((16,(14,15)),((9,(7,(6,8))),(13,((11,(10,12)),(5,(4,(0,(3,(1,2)))))))))));
		ML	(18,((17,(16,(14,15))),((13,(4,(0,(2,(1,3))))),(12,((9,(10,11)),(5,(6,(7,8))))))));

Table S1: Vibrionaceae 19–taxon large chromosome dataset LCBs and trees (continued).

LCB	Aligned bp	Algorithm	Tree topologies (Newick)
113	1,132	MP	(18,((17,(15,16)),(14,(5,(13,((9,(7,(6,8))),((11,(10,12)),(4,(0,(3,(1,2))))))))))));
		ML	(18,((17,(12,((7,8),(5,6)))),((4,(13,(16,(14,15)))),((11,(9,10)),(0,(2,1,3))))));
114	14,588	MP	(18,((16,(14,15)),(17,((13,(11,12)),((8,(6,(7,9))),(10,(5,(4,(1,(3,(2,0))))))))))));
		ML	(18,(17,((16,(14,15)),(12,((4,(13,(0,(3,2,1)))),((10,(9,11)),(5,(6,(7,8)))))))));
115	32,461	MP	(18,(17,((16,(14,15)),(13,((12,(11,(9,10))),((8,(6,7)),(5,(4,(0,(2,(1,3)))))))))));
		ML	(18,((17,(16,(14,15))),((4,(13,(0,(2,(1,3))))),(12,((10,(9,11)),(5,(7,(6,8))))))));
116	70,298	MP	(18,(17,((16,(14,15)),((0,(2,(1,3))),((12,(10,11)),(9,(7,(8,(6,(13,(4,5)))))))))));
		ML	(18,((17,(16,(14,15))),((4,(13,(0,(1,2,3)))),(12,((11,(9,10)),(5,(8,(6,7))))))));
117	12,581	MP	(18,(17,((1,(2,(3,0))),((11,(9,10)),(13,((16,(14,15)),(6,(7,(8,(5,(4,12)))))))))));
		ML	(18,((17,(16,(14,15))),((4,(13,(0,(3,2,1)))),(12,((9,(10,11)),(5,(6,(7,8))))))));
118	22,480	MP	(18,(14,((17,(15,16)),((11,(10,12)),((9,(8,(6,7))),(13,(5,(4,(0,(3,(1,2)))))))))));
		MP	(18,(4,((13,(11,12)),(14,(((7,8),(9,10)),((17,(15,16)),(6,(5,(0,(1,(2,3)))))))))));
		ML	(18,(17,((16,(14,15)),((4,(13,(2,0,(1,3)))),(12,((10,(9,11)),(5,(8,(6,7)))))))));
119	2,810	MP	(18,(17,((16,(14,15)),(4,((0,(3,(1,2))),(13,(9,(8,((7,(5,6)),(12,(10,11)))))))))));
		ML	(18,(17,((16,(14,15)),(12,((11,(9,10)),((5,(8,(6,7))),(4,(13,(0,1,3,2)))))))));
120	8,667	MP	(18,(17,((16,(14,15)),((3,(1,(2,0))),(4,(5,(13,((12,(10,11)),(9,(8,(6,7)))))))))));
		ML	(18,((17,(16,(14,15))),(12,((4,(13,(0,2,3,1))),((9,(10,11)),(8,(5,(6,7))))))));
121	14,205	MP	(18,(13,(17,((16,(14,15)),(12,((5,(8,(6,7))),((11,(9,10)),(4,(0,(1,(2,3)))))))))));
		ML	(18,(13,((0,(3,1,2)),(4,(12,((17,(16,(14,15))),((10,(9,11)),((6,8),(5,7)))))))));
122	13,045	MP	(18,((16,(14,15)),(9,(5,((0,(1,(2,3))),(4,((8,(6,7)),(17,(13,(11,(10,12)))))))))));
		ML	(18,(12,(17,(16,(14,15))),((4,(13,(0,(2,(1,3))))),((9,(10,11)),(5,(7,(6,8)))))));
123	16,276	MP	(18,(17,((16,(14,15)),((12,(10,11)),((6,(7,(8,9))),(13,(5,(4,(3,(2,(1,0)))))))))));
		ML	(18,(12,((17,(16,(14,15))),(5,(7,(6,8)))),(4,((13,(10,(9,11))),(0,(3,1,2))))));
124	4,509	MP	(18,(17,((15,16),(14,(13,((5,(4,(0,(1,(2,3))))),((12,(10,11)),(9,(7,(6,8))))))))));
		ML	(18,(((4,16),(17,(13,(0,(2,1,3))))),((12,(14,15)),((9,(10,11)),(5,(6,(7,8)))))));
125	2,681	MP	(18,(((2,3),(1,0)),(4,(13,(17,(12,((7,(6,8)),(5,((10,(9,11)),(16,(14,15)))))))))));
		ML	(18,(17,(16,(14,15)),((13,(2,0,1,3)),((10,(9,11)),((4,12),(5,(7,(6,8))))))));
126	15,355	MP	(18,(13,((10,(8,9)),(14,((0,(3,(1,2))),(12,(11,((15,(16,17)),(4,(7,(5,6)))))))))));
		ML	(18,(17,((16,(14,15)),(((11,(9,10)),(5,(6,(7,8)))),(12,(4,(13,(0,(2,3,1)))))))));
127	4,661	MP	(18,(14,(4,(5,(10,((6,(7,8)),((17,(15,16)),((9,(13,(11,12))),((1,2),(3,0))))))))));
		ML	(18,(13,((0,(3,1,2)),(4,((17,(16,(14,15))),(12,((10,(9,11)),(5,(8,(6,7))))))))));
128	1,950	MP	(18,(16,((15,(13,14)),(12,((5,(8,(6,7))),((9,(10,11)),(4,(17,(2,(1,(3,0)))))))))));
		ML	(18,(17,((16,(14,15)),(12,(8,((5,(6,7)),((10,(9,11)),(4,(13,(0,(1,2,3))))))))));
129	10,960	MP	(18,((17,(15,16)),(13,((11,(9,10)),((7,(8,(5,6))),(12,(14,(4,(0,(1,(2,3)))))))))));
		MP	(18,(17,((16,(14,15)),(13,((9,(8,(6,7))),((12,(10,11)),(5,(4,(0,(3,(1,2)))))))))));
		ML	(18,(17,(13,((0,(1,3,2)),(4,((12,(16,(14,15))),((11,(9,10)),(5,(8,(6,7))))))))));
130	4,163	MP	(18,((5,(6,(7,8))),(15,(16,((12,(10,11)),(9,((17,(13,14)),(4,(3,(1,(2,0)))))))))));
		ML	(18,((13,(0,(2,(1,3)))),(4,((12,(17,(16,(14,15)))),((10,(9,11)),(5,(8,(6,7))))))));
131	1,545	MP	(18,(17,(16,(4,(((1,2),(3,0)),(5,((13,(14,15)),((12,(10,11)),(6,(7,(8,9)))))))))));

Table S1: Vibrionaceae 19–taxon large chromosome dataset LCBs and trees (continued).

LCB	Aligned bp	Algorithm	Tree topologies (Newick)
		ML	(18,(4,((13,(0,(2,(1,3)))),((17,(16,(14,15))),(12,((10,(9,11)),(5,(6,(7,8)))))))));
132	2,576	MP	(18,(9,((12,(10,11)),(16,((0,(2,(1,3))),(17,((13,(14,15)),(8,(4,(5,(6,7))))))))));
		ML	(18,((17,(16,(14,15))),(((10,(9,11)),(5,(8,(6,7)))),(12,(4,(13,(0,(2,3,1))))))));
133	2,054	MP	(18,((17,(15,(13,14))),(7,((4,(10,(8,9))),((5,6),(16,(11,(12,(0,(3,(1,2))))))))));
		ML	(18,(17,((16,(14,15)),(12,((4,(13,(0,(3,2,1)))),((9,(10,11)),(5,(8,(6,7)))))))));
134	13,070	MP	(18,(17,((14,(12,13)),(16,((0,(2,(1,3))),(15,(11,((10,(8,9)),(4,(5,(6,7)))))))))));
		MP	(18,((17,(16,(14,15))),(13,((10,(8,9)),(7,((4,(5,6)),(11,(12,(0,(1,(2,3))))))))));
		ML	(18,(17,((16,(14,15)),((0,3,2,1),(4,((12,13),((9,(10,11)),(5,(7,(6,8))))))))));
135	6,032	MP	(18,(17,((16,(14,15)),(11,((4,(5,(6,7))),((10,(8,9)),(12,(13,(0,(2,(1,3)))))))))));
		MP	(18,(17,((15,16),(14,(13,((5,(4,((1,2),(3,0)))),((10,(11,12)),(9,(8,(6,7)))))))))));
		ML	(18,((17,(16,(14,15))),(((9,(10,11)),(8,(6,(5,7)))),(12,(4,(13,(2,0,1,3)))))));
136	3,416	MP	(18,((16,(14,15)),(4,(8,((7,(5,6)),((9,17),((13,(11,12)),(10,((2,3),(1,0))))))))));
		ML	(18,(17,((16,(14,15)),((12,(10,(9,11))),((5,(8,(6,7))),(4,(13,(0,1,2,3))))))));
137	5,412	MP	(18,(16,(17,((15,(13,14)),(12,((11,(0,(3,(1,2)))),((10,(8,9)),(7,(6,(4,5))))))))));
		ML	(18,((0,3,2,1),(13,(4,((12,(17,(16,(14,15)))),((10,(9,11)),(5,(7,(6,8)))))))));
138	2,987	MP	(18,((11,((4,5),(10,(8,9)))),((15,(13,14)),(17,(16,(12,(7,(6,((2,3),(1,0))))))))));
		ML	(18,(4,((13,(0,3,1,2)),((17,(16,(14,15))),(12,((11,(9,10)),((7,8),(5,6))))))));
139	2,730	MP	(18,(17,((16,(14,15)),(13,((5,(4,(0,(3,(1,2))))),((11,(10,12)),(8,(9,(6,7)))))))));
		ML	(18,(4,((3,1,0,2),(13,((17,(16,(14,15))),(12,((10,(9,11)),(5,(6,(7,8))))))))));
140	7,943	MP	(18,(17,((16,(14,15)),(13,((4,(5,(0,(2,(1,3))))),((12,(10,11)),(6,(8,(7,9)))))))));
		ML	(18,((0,2,1,3),(13,(4,((12,(17,(14,(15,16)))),((9,(10,11)),(8,(5,(6,7)))))))));
141	6,310	MP	(18,(17,((16,(14,15)),(13,((5,(4,(0,(3,(1,2))))),((12,(10,11)),(9,(6,(7,8)))))))));
		ML	(18,(17,((16,(14,15)),(12,((4,(13,(0,1,3,2))),((9,(10,11)),(5,(8,(6,7)))))))));
142	2,069	MP	(18,(17,((16,(14,15)),(13,((5,(4,(0,(1,(2,3))))),((12,(10,11)),((6,7),(8,9))))))));
		ML	(18,(4,(13,((2,1,0,3),(12,((7,(5,(6,8))),((9,(10,11)),(17,(16,(14,15))))))))));
143	12,239	MP	(18,(16,((8,(6,7)),((4,(0,(3,(1,2)))),(5,(17,((13,(14,15)),(9,(10,(11,12))))))))));
		ML	(18,((17,(16,(14,15))),((4,(13,(1,3,0,2))),(12,((9,(10,11)),(8,(5,(6,7))))))));
144	12,487	MP	(18,(17,((16,(14,15)),(12,(13,((4,(0,(1,(2,3)))),((10,(9,11)),(5,(8,(6,7))))))))));
		ML	(18,(17,((16,(14,15)),(((9,10,11),(7,(5,(6,8)))),(12,(4,(13,(0,2,1,3))))))));
145	5,498	MP	(18,(13,((16,(14,15)),(17,(8,((4,(0,(2,(1,3)))),((5,(6,7)),(9,(12,(10,11))))))))));
		ML	(18,((12,(4,(13,(2,(0,(1,3)))))),((17,(16,(14,15))),((9,(10,11)),(5,(8,(6,7)))))));
146	4,223	MP	(18,((16,(14,15)),(17,(10,((0,(2,(1,3))),((11,(12,13)),((4,5),(6,(9,(7,8))))))))));
		ML	(18,(17,((16,(14,15)),((4,(13,(0,2,1,3))),(12,((9,(10,11)),(5,(8,(6,7)))))))));
147	6,886	MP	(18,((17,(16,(14,15))),(13,((6,(8,(7,9))),((12,(10,11)),(5,(4,(3,(1,(2,0))))))))));
		ML	(18,((17,(16,(14,15))),((4,(13,(0,(3,1,2)))),(12,((11,(9,10)),(5,(6,(7,8))))))));
148	5,239	MP	(18,((13,(11,(9,10))),(12,(((1,2),(3,0)),(17,((14,(15,16)),(7,(8,(4,(5,6))))))))));
		ML	(18,((17,(16,(14,15))),((4,(13,(0,(3,1,2)))),(12,((10,(9,11)),(7,(6,(5,8))))))));
149	7,632	MP	(18,((17,(16,(14,15))),((0,(2,(1,3))),(4,(12,(13,((9,(10,11)),(8,(5,(6,7))))))))));
		ML	(18,((17,(16,(14,15))),(12,((4,(13,(3,0,2,1))),((9,(10,11)),(5,(8,(6,7))))))));

Table S1: Vibrionaceae 19–taxon large chromosome dataset LCBs and trees (continued).

LCB	Aligned bp	Algorithm	Tree topologies (Newick)
150	17,681	MP	(18,(17,((16,(14,15)),(10,((5,(4,(0,(2,(1,3))))),((12,(11,13)),(6,(7,(8,9)))))))));
		ML	(18,((17,(16,(14,15))),((4,(13,(0,(2,(1,3))))),(12,((10,(9,11)),(5,(7,(6,8))))))));
151	3,564	MP	(18,(((4,(5,6)),(12,(11,(9,10)))),(17,((16,(14,15)),(13,(7,(8,(3,(1,(2,0)))))))))));
		MP	(18,(17,((16,(14,15)),((12,(10,11)),((13,(6,(8,(7,9)))),(5,(4,(1,(0,(2,3))))))))));
		ML	(18,((17,(16,(14,15))),((8,(12,(5,(6,7)))),((10,(9,11)),(13,(4,(0,3,2,1)))))));
152	4,675	MP	(18,(17,((16,(14,15)),(12,((13,(4,(0,(2,(1,3))))),((9,(10,11)),(5,(6,(7,8)))))))));
		MP	(18,(8,(16,((11,(9,10)),(17,((15,(4,(5,(6,7)))),((14,(12,13)),(2,(1,(3,0))))))))));
		ML	(18,((17,(16,(14,15))),(12,((4,(13,(0,(3,1,2)))),((11,(9,10)),(5,(8,(6,7))))))));
153	21,898	MP	(18,((17,(16,(14,15))),(12,(((1,3),(2,0)),(13,(4,((11,(9,10)),(5,(7,(6,8))))))))));
		ML	(18,(17,((16,(14,15)),(12,((4,(13,(0,(2,(1,3))))),((11,(9,10)),(5,(8,(6,7)))))))));
154	8,162	MP	(18,((17,(15,16)),(14,(12,((13,(11,(1,(3,(2,0))))),((8,(9,10)),(7,(6,(4,5)))))))));
		ML	(18,((17,(16,(14,15))),((4,(13,(0,(3,2,1)))),(12,((9,(10,11)),(7,(8,(5,6))))))));
155	12,301	MP	(18,(13,((17,(16,(14,15))),((9,(8,10)),((7,(5,(4,6))),(12,(11,(0,(2,(1,3))))))))));
		ML	(18,((12,(4,(13,(0,(2,(1,3)))))),((17,(16,(14,15))),((9,(10,11)),(5,(6,(7,8)))))));
156	6,664	MP	(18,((7,(4,(5,6))),((17,(15,16)),(13,((0,(2,(1,3))),(8,(12,(14,(11,(9,10))))))))));
		ML	(18,((17,(16,(14,15))),((4,(13,(0,(3,1,2)))),(12,((10,(9,11)),(8,(5,(6,7))))))));
157	5,630	MP	(18,((14,(17,(15,16))),(10,((9,(2,(1,(3,0)))),(8,(7,((5,6),(4,(13,(11,12))))))))));
		ML	(18,(0,(3,1,2,(4,(13,((17,(16,(14,15))),(12,((11,(9,10)),(5,(8,(6,7))))))))));
158	7,687	MP	(18,(9,(8,(13,((17,(16,(14,15))),((2,(3,(1,0))),((11,(10,12)),(4,(5,(6,7))))))))));
		ML	(18,(6,(8,(5,(7,((10,(9,11)),(12,(17,((16,(14,15)),(4,(13,(3,(2,1,0))))))))))));
159	23,214	MP	(18,((10,(8,9)),((12,(3,(2,(1,0)))),(17,(6,(7,((5,(4,11)),(16,(15,(13,14))))))))));
		ML	(18,((17,(16,(14,15))),(12,((4,(13,(0,(3,2,1)))),((11,(9,10)),((6,8),(5,7)))))));
160	7,274	MP	(18,(17,((16,(14,15)),(13,((9,(6,(7,8))),((12,(10,11)),((4,5),(3,(0,(1,2))))))))));
		ML	(18,((1,0,2,3),((4,13),((10,(9,11)),((6,(7,8)),(5,(12,(17,(16,(14,15))))))))));
161	7,872	MP	(18,(17,((7,(5,6)),((0,(3,(4,(1,2)))),(8,(12,((9,(10,11)),(13,(16,(14,15))))))))));
		ML	(18,(17,((16,(14,15)),(4,((12,(5,(8,(6,7)))),((11,(9,10)),(13,(1,0,2,3))))))));
162	17,207	MP	(18,(17,((0,(3,(1,2))),(12,(13,((11,(16,(14,15))),((8,(9,10)),(7,(4,(5,6))))))))));
		MP	(18,(17,((16,(14,15)),(8,((13,(12,(0,(3,(1,2))))),((11,(9,10)),(4,(7,(5,6)))))))));
		MP	(18,((16,(14,15)),(17,(12,((13,(4,(0,(2,(1,3))))),((11,(9,10)),(8,(7,(5,6)))))))));
		ML	(18,((16,(14,15)),(17,((8,(5,(6,7))),(12,((9,(10,11)),(4,(13,(0,2,(1,3))))))))));
163	7,476	MP	(18,(14,((17,(15,16)),(13,((4,(5,(2,(3,(1,0))))),((11,(10,12)),(6,(8,(7,9)))))))));
		ML	(18,(((10,(9,11)),(5,(6,(7,8)))),(12,((17,(16,(14,15))),(4,(13,(2,0,3,1)))))));
164	17,343	MP	(18,(17,((16,(14,15)),(10,(8,((9,((2,3),(1,0))),((11,(12,13)),(4,(6,(5,7))))))))));
		ML	(18,(17,((16,(14,15)),((4,(13,(0,(3,2,1)))),(12,((9,(10,11)),(5,(8,(6,7)))))))));
165	4,416	MP	(18,((16,(14,15)),(17,(12,((13,(4,(2,(3,(1,0))))),((11,(9,10)),(8,(7,(5,6)))))))));
		ML	(18,(17,((16,(14,15)),((4,(13,(0,1,3,2))),(12,((11,(9,10)),(5,(6,(7,8)))))))));
166	4,744	MP	(18,(17,((16,(14,15)),(5,((3,(2,(1,0))),((6,(7,8)),((4,13),(9,(12,(10,11))))))))));
		ML	(18,(13,(0,3,1,2),(4,((17,(16,(14,15))),(12,((9,(10,11)),(5,(6,(7,8)))))))));
167	9,449	MP	(18,(5,(((1,2),(3,0)),(4,((14,(17,(15,16))),(13,(6,((7,(8,9)),(12,(10,11))))))))));

Table S1: Vibrionaceae 19–taxon large chromosome dataset LCBs and trees (continued).

LCB	Aligned bp	Algorithm	Tree topologies (Newick)
		ML	(18,((4,(13,(0,2,(1,3)))),((17,(16,(14,15))),(12,((9,(10,11)),(5,(7,(6,8))))))));
168	34,043	MP	(18,(11,((13,14),(17,((15,(3,(0,(1,2)))),(12,((9,(8,10)),((7,16),(4,(5,6))))))))));
		MP	(18,((16,(14,15)),(17,(13,((5,(4,(0,(1,(2,3))))),((12,(10,11)),(6,(9,(7,8)))))))));
		MP	(18,((17,(16,(14,15))),((9,(7,(8,10))),((11,(12,13)),(6,(5,(4,(0,(3,(1,2))))))))));
		ML	(18,((4,(13,(0,2,(1,3)))),((17,(16,(14,15))),(12,((9,(10,11)),(5,(7,(6,8))))))));
169	17,574	MP	(18,((16,17),((14,15),(11,((12,(13,(0,(3,(1,2))))),((8,(9,10)),(4,(7,(5,6)))))))));
		ML	(18,(17,((16,(14,15)),(12,((4,(13,(0,(2,(1,3))))),((9,(10,11)),(5,(8,(6,7)))))))));
170	1,685	MP	(18,((16,(14,15)),(17,(13,((5,(4,(0,(3,(1,2))))),((12,(10,11)),(9,(7,(6,8)))))))));
		ML	(18,(17,((16,(14,15)),(((5,8),(6,7)),(12,((11,(9,10)),(4,(13,(0,3,1,2)))))))));
171	15,568	MP	(18,(14,((17,(15,16)),(13,((12,(11,((1,3),(2,0)))),((5,7),(6,(4,(8,(9,10))))))))));
		ML	(18,((17,(16,(14,15))),(12,((4,(13,(0,(2,3,1)))),((10,(9,11)),(8,(5,(6,7))))))));
172	4,279	MP	(18,(17,((13,(11,12)),(14,(15,((4,(5,(6,7))),((8,(9,10)),(16,((2,3),(1,0))))))))));
		ML	(18,(17,((16,(14,15)),(12,((4,13),((8,(7,(5,6))),((11,10,9),(0,1,3,2))))))));
173	2,574	MP	(18,(4,((0,(2,(1,3))),(13,((17,(16,(14,15))),(12,((11,(9,10)),(8,(5,(6,7))))))))));
		ML	(18,(17,(16,(14,15)),(12,((4,(13,(0,2,3,1))),((10,(9,11)),(8,(7,(5,6))))))));
174	30,294	MP	(18,((17,(16,(14,15))),(13,((12,(10,11)),((6,(7,(8,9))),(5,(4,(3,(2,(1,0))))))))));
		ML	(18,(17,((16,(14,15)),((4,(13,(2,(0,1,3)))),(12,((9,(10,11)),(5,(8,(6,7)))))))));
175	4,739	MP	(18,((17,((13,14),(15,16))),(12,((7,(5,6)),((11,(9,10)),(4,(8,(0,(1,(2,3))))))))));
		ML	(18,(4,(13,(0,2,(1,3))),((12,(17,(16,(14,15)))),((10,(9,11)),(5,(6,(7,8)))))));
176	8,924	MP	(18,(17,((16,(14,15)),(13,((5,(4,(0,(3,(1,2))))),((11,(10,12)),(9,(8,(6,7)))))))));
		ML	(18,(17,((16,(14,15)),(4,((13,(0,1,2,3)),((11,(9,10)),(12,(8,(5,(6,7))))))))));
177	11,027	MP	(18,(17,((16,(14,15)),(4,((5,(0,(3,(1,2)))),(13,((12,(10,11)),(6,(9,(7,8))))))))));
		ML	(18,((4,(13,(0,(2,1,3)))),((17,(16,(14,15))),(12,((10,(9,11)),(6,(5,(7,8))))))));
178	53,816	MP	(18,((16,(14,15)),(17,((0,(2,(1,3))),(13,(10,((4,(5,6)),((11,12),(9,(7,8)))))))));
		ML	(18,((17,(16,(14,15))),((4,(13,(0,(2,(1,3))))),(12,((9,(10,11)),(5,(7,(6,8))))))));
179	4,791	MP	(18,((17,(15,(13,14))),(5,(4,((1,(0,(2,3))),(16,((11,(10,12)),(9,(6,(7,8))))))))));
		ML	(18,(10,(11,(9,((12,(4,(13,(0,2,3,1)))),((17,(16,(14,15))),(5,(6,(7,8)))))))));
180	11,262	MP	(18,(13,((4,(0,(2,(1,3)))),(12,(17,((16,(14,15)),((11,(9,10)),(5,(6,(7,8))))))))));
		ML	(18,(17,((16,(14,15)),(12,((8,(6,(5,7))),((10,(9,11)),(4,(13,(0,(3,2,1))))))))));
181	5,463	MP	(18,(17,((16,(14,15)),(12,((13,(4,(0,(1,(2,3))))),((10,(9,11)),(5,(6,(7,8)))))))));
		ML	(18,(13,((4,(0,1,3,2)),((12,(17,(16,(14,15)))),((10,(9,11)),(6,(8,(5,7))))))));
182	28,543	MP	(18,(4,((0,(2,(1,3))),((16,(14,15)),((13,17),(12,((9,(10,11)),(5,(6,(7,8))))))))));
		ML	(18,(17,((16,(14,15)),((4,(13,(0,2,1,3))),(12,((11,(9,10)),(5,(8,(6,7)))))))));
183	8,107	MP	(18,(13,(17,((8,(9,(6,(5,7)))),((4,(0,(3,(1,2)))),(16,(15,(14,(11,(10,12))))))))));
		ML	(18,(17,((12,(16,(14,15))),(((5,8),(6,7)),((11,(9,10)),(4,(13,(0,2,1,3))))))));
184	4,562	MP	(18,(17,(16,((14,15),((10,(9,11)),((12,(4,(5,(6,7)))),((8,13),(0,(3,(1,2))))))))));
		MP	(18,((16,(15,(14,17))),(9,((5,(8,(6,7))),(13,(4,((12,(10,11)),(0,(2,(1,3))))))))));
		ML	(18,((17,(16,(14,15))),((4,(13,(0,(2,1,3)))),(12,((10,(9,11)),(8,(7,(5,6))))))));
185	39,671	MP	(18,(9,(17,((0,(3,(1,2))),((14,(15,16)),((4,(5,(6,7))),(8,(13,(12,(10,11))))))))));

Table S1: Vibrionaceae 19–taxon large chromosome dataset LCBs and trees (continued).

LCB	Aligned bp	Algorithm	Tree topologies (Newick)
		MP	(18,((14,(17,(15,16))),((4,(0,(1,(2,3)))),(13,(8,((6,7),(5,(9,(10,(11,12))))))))));
		ML	(18,(17,((16,(14,15)),((13,(4,(2,0,(1,3)))),(12,((11,(9,10)),(5,(7,(6,8)))))))));
186	28,901	MP	(18,((16,(14,15)),(17,(12,(((5,6),(7,8)),((11,(9,10)),(13,(4,((2,3),(1,0))))))))));
		ML	(18,(17,((16,(14,15)),(12,((4,(13,(0,(2,(1,3))))),((9,(10,11)),(5,(6,(7,8)))))))));
187	7,952	MP	(18,(17,((16,(14,15)),(12,((13,(4,(0,(3,(1,2))))),((11,(9,10)),(8,(5,(6,7)))))))));
		ML	(18,(17,((16,(14,15)),(12,((4,(13,(0,(2,(1,3))))),((10,(9,11)),((5,7),(6,8))))))));
188	20,749	MP	(18,(16,((5,8),(4,(17,(((10,11),(9,(12,(6,7)))),((15,(13,14)),((2,3),(1,0)))))))));
		ML	(18,((4,(13,(0,(2,(1,3))))),((12,(17,(16,(14,15)))),((10,(9,11)),(5,(8,(6,7)))))));
189	2,134	MP	(18,(17,((14,(12,13)),((11,(4,(7,(5,6)))),((9,(8,10)),(15,(16,(0,(3,(1,2))))))))));
		ML	(18,(((4,13),(0,1,2,3)),(17,((16,(14,15)),(12,((11,(9,10)),(5,(7,(6,8)))))))));
190	23,069	MP	(18,((14,(17,(15,16))),(11,(((1,3),(2,0)),(12,(13,((10,(8,9)),(7,(4,(5,6))))))))));
		ML	(18,((0,(1,2,3)),(13,(4,((12,(17,(16,(14,15)))),((10,(9,11)),(8,(5,(6,7)))))))));
191	29,839	MP	(18,((13,(16,17)),(14,(12,(15,((4,(0,(3,(1,2)))),((8,(6,7)),(5,(11,(9,10))))))))));
		ML	(18,((17,(16,(14,15))),(12,((4,(13,(0,(3,1,2)))),((10,(9,11)),(5,(8,(6,7))))))));
192	5,354	MP	(18,(16,((17,(15,(13,14))),((0,(3,(1,2))),(4,(5,((10,(11,12)),(6,(9,(7,8))))))))));
		ML	(18,((17,(12,(16,(14,15)))),(5,((6,(7,8)),(13,(4,((11,(9,10)),((1,3),(0,2)))))))));
193	33,253	MP	(18,((10,(8,9)),(11,(15,((16,(17,(0,(3,(1,2))))),((14,(12,13)),(6,(7,(4,5)))))))));
		ML	(18,((17,(16,(14,15))),(12,((4,(13,((1,3),(0,2)))),((10,(9,11)),(5,(7,(6,8))))))));
194	5,802	MP	(18,((16,17),((14,15),(13,((5,(4,(0,(2,(1,3))))),((12,(10,11)),(9,(7,(6,8)))))))));
		ML	(18,(17,((16,(14,15)),(12,((4,(13,(3,1,0,2))),((10,(9,11)),(5,(8,(6,7)))))))));
195	26,196	MP	(18,((4,17),((16,(14,15)),(13,(12,((0,(3,(1,2))),((6,(5,7)),(8,(10,(9,11))))))))));
		ML	(18,(7,(5,6),(8,((11,(9,10)),((12,(17,(16,(14,15)))),(4,(13,(0,(3,1,2)))))))));
196	6,517	MP	(18,(17,((16,(14,15)),(((11,(10,12)),(9,(6,(7,8)))),(13,(5,(4,(0,(3,(1,2))))))))));
		ML	(18,((17,(16,(14,15))),((4,(13,(0,(2,3,1)))),(12,((10,(9,11)),(5,(6,(7,8))))))));
197	9,422	MP	(18,(17,((16,(14,15)),(13,((5,(4,(3,(0,(1,2))))),((11,(10,12)),(6,(7,(8,9)))))))));
		ML	(18,(13,((0,3,1,2),((10,(9,11)),(4,((8,(7,(5,6))),(12,(17,(16,(14,15))))))))));
198	5,196	MP	(18,(17,((16,(14,15)),(13,((5,(4,(0,(1,(2,3))))),((12,(10,11)),(9,(6,(7,8)))))))));
		ML	(18,(17,(12,(16,(14,15))),((4,(13,(1,2,0,3))),((9,(10,11)),((5,6),(7,8))))));
199	10,917	MP	(18,((17,(16,(14,15))),((12,(0,(1,(2,3)))),((9,10),(11,(4,(13,(5,(6,(7,8))))))))));
		ML	(18,(17,((16,(14,15)),((4,(13,(1,0,3,2))),(12,((9,(10,11)),(5,(8,(6,7)))))))));
200	9,889	MP	(18,((16,17),(15,(14,((5,(4,(0,(3,(1,2))))),(13,((12,(10,11)),(9,(6,(7,8))))))))));
		ML	(18,(17,((16,(14,15)),(12,(5,((7,(6,8)),((9,(10,11)),(4,(13,(0,(2,(1,3)))))))))))
201	23,891	MP	(18,(17,((16,(14,15)),(13,((5,(4,(0,(1,(2,3))))),((10,(11,12)),(6,(9,(7,8)))))))));
		ML	(18,(17,((16,(14,15)),(12,((4,(13,(0,(2,3,1)))),((11,(9,10)),(5,(6,(7,8)))))))));
202	848	MP	(18,(17,((16,(14,15)),(12,((13,(4,(3,(0,(1,2))))),((9,(10,11)),(5,(8,(6,7)))))))));
		ML	(18,(17,((16,(14,15)),(12,((4,(13,(0,(2,(1,3))))),((10,9,11),(6,(5,(7,8)))))))));
203	8,067	MP	(18,(17,((16,(14,15)),(((11,(10,12)),(9,(8,(6,7)))),(13,(5,(4,(0,(1,(2,3))))))))));
		ML	(18,(((1,3),(0,2)),(13,(4,((10,(9,11)),((8,(6,7)),(5,(12,(17,(16,(14,15)))))))))));
204	4,838	MP	(18,(17,((16,(14,15)),(13,((5,(4,(0,(3,(1,2))))),((12,(10,11)),(9,(6,(7,8)))))))));

Table S1: Vibrionaceae 19–taxon large chromosome dataset LCBs and trees (continued).

LCB	Aligned bp	Algorithm	Tree topologies (Newick)
		ML	(18,(17,((16,(14,15)),(12,((4,(13,(3,1,(0,2)))),((11,(9,10)),((7,8),(5,6))))))));
205	9,436	MP	(18,(17,((16,(14,15)),((5,(4,((2,3),(1,0)))),(13,((12,(10,11)),(6,(9,(7,8)))))))));
		MP	(18,((14,(17,(15,16))),(13,((11,(12,(0,(3,(1,2))))),((8,(9,10)),((4,7),(5,6)))))));
		ML	(18,((17,(16,(14,15))),(12,((4,(13,(0,(2,(1,3))))),((10,(9,11)),(5,(8,(6,7))))))));
206	11,145	MP	(18,((13,((12,(10,11)),(4,(7,(5,6))))),(17,((16,(14,15)),(8,(9,(0,(3,(1,2)))))))));
		ML	(18,((17,(16,(14,15))),((4,(13,(0,(3,1,2)))),(12,((11,(9,10)),(8,(7,(5,6))))))));
207	15,746	MP	(18,(((15,(16,17)),(11,(12,(13,14)))),((4,7),((10,(8,9)),(6,(5,(0,(1,(2,3)))))))));
		ML	(18,((17,(16,(14,15))),((4,(13,(0,(2,1,3)))),(12,((11,(9,10)),(5,(7,(6,8))))))));
208	7,339	MP	(18,(17,((16,(14,15)),((5,(4,(0,(3,(1,2))))),(13,((12,(10,11)),(9,(6,(7,8)))))))));
		ML	(18,(17,((16,(14,15)),((4,(13,(0,(1,2,3)))),(12,((11,(9,10)),(5,(7,(6,8)))))))));
209	22,240	MP	(18,(17,((16,(14,15)),((5,(4,(0,(3,(1,2))))),(13,((12,(10,11)),(9,(8,(6,7)))))))));
		ML	(18,(17,((16,(14,15)),((4,(13,(0,(3,1,2)))),(12,((10,(9,11)),(5,(7,(6,8)))))))));
210	17,557	MP	(18,((17,(16,(14,15))),(9,((5,(7,(6,8))),((13,(11,12)),(10,(4,((1,3),(2,0)))))))));
		ML	(18,((17,(16,(14,15))),((4,(13,(0,(2,(1,3))))),(12,((11,(9,10)),(5,(7,(6,8))))))));
211	6,211	MP	(18,((17,(16,(14,15))),((13,(9,(8,(6,7)))),(5,(4,((12,(10,11)),(0,(3,(1,2)))))))));
		MP	(18,((16,(14,15)),(17,((5,(4,(0,(2,(1,3))))),(13,((12,(10,11)),(9,(6,(7,8)))))))));
		ML	(18,(17,((16,(14,15)),((4,(13,(0,(3,1,2)))),(12,((10,(9,11)),(5,(8,(6,7)))))))));
212	847	MP	(18,(17,((13,14),((4,(15,16)),((12,(0,(2,(1,3)))),((9,(10,11)),((5,6),(7,8))))))));
		ML	(18,((17,(16,(14,15))),((11,(9,10)),(12,(5,(7,(6,8)))),(4,(13,(3,1,0,2))))));
213	2,377	MP	(18,((17,(16,(14,15))),(13,((5,(4,(0,(3,(1,2))))),((10,(11,12)),(6,(8,(7,9))))))));
		ML	(18,(17,((16,(14,15)),(12,((5,(6,(7,8))),((10,(9,11)),(4,(13,(3,0,2,1)))))))));
214	3,380	MP	(18,(11,((12,(0,(1,(2,3)))),(13,((17,(16,(14,15))),((8,(9,10)),(7,(4,(5,6)))))))));
		ML	(18,(17,((16,(14,15)),(4,((13,(3,0,2,1)),(12,((11,(9,10)),(5,(7,(6,8))))))))));
215	11,992	MP	(18,((16,17),((6,(5,(4,(0,(2,(1,3)))))),(15,(14,((12,(10,11)),(13,(9,(7,8)))))))));
		ML	(18,((13,(2,3,0,1)),((4,(12,(17,(16,(14,15))))),((11,(9,10)),(7,(8,(5,6)))))));
216	11,235	MP	(18,(6,((8,(7,(5,(17,(12,(10,11)))))),(9,(4,((16,(14,15)),(13,((2,3),(1,0)))))))));
		ML	(18,((17,(16,(14,15))),(((11,(9,10)),(5,(6,(7,8)))),(12,(4,(13,(0,(1,3,2))))))));
217	4,205	MP	(18,(16,(11,(((4,(5,6)),(10,(8,9))),((7,(13,(17,(14,15)))),(12,((2,3),(1,0))))))));
		ML	(18,((13,(0,(3,1,2))),(4,((12,((16,17),(14,15))),((10,(9,11)),(8,(7,(5,6))))))));
218	8,844	MP	(18,((17,(15,16)),(14,(13,((5,(4,((1,3),(2,0)))),((10,(11,12)),(6,(9,(7,8)))))))));
		ML	(18,((17,(16,(14,15))),(12,((4,(13,(0,(3,2,1)))),((10,(9,11)),(5,(8,(6,7))))))));
219	18,577	MP	(18,(17,(((4,(16,(14,15))),((1,3),(2,0))),(12,((11,(9,10)),(13,(5,(8,(6,7)))))))));
		ML	(18,((17,(16,(14,15))),(((9,(10,11)),(5,(7,(6,8)))),(12,(4,(13,(0,(2,(1,3)))))))));
220	1,632	MP	(18,(9,(13,((17,(16,(14,15))),((4,((2,3),(1,0))),((11,(10,12)),(8,(7,(5,6)))))))));
		MP	(18,(12,((4,(11,(9,10))),(((5,6),(7,8)),((14,(17,(15,16))),(13,(0,(1,(2,3)))))))));
		ML	(18,(13,(0,(2,1,3)),(4,((12,(17,(16,(14,15)))),((9,(10,11)),((6,8),(5,7)))))));
221	4,526	MP	(18,((2,(3,(1,0))),(11,(12,((16,(17,(15,(13,14)))),((8,(9,10)),((4,5),(6,7))))))));
		ML	(18,((9,(10,11)),(5,(8,(6,7))),(12,((4,(17,(16,(14,15)))),(13,(0,(1,2,3)))))));
222	17,101	MP	(18,((17,(16,(14,15))),(13,((5,(4,(0,(3,(1,2))))),((12,(10,11)),(6,(8,(7,9))))))));

Table S1: Vibrionaceae 19–taxon large chromosome dataset LCBs and trees (continued).

LCB	Aligned bp	Algorithm	Tree topologies (Newick)
		ML	(18,((4,(13,(0,(3,2,1)))),((12,(17,(16,(14,15)))),((9,(10,11)),(5,(8,(6,7)))))));
223	6,326	MP	(18,((16,17),((14,15),((13,(12,(0,(2,(1,3))))),(((4,7),(5,6)),(11,(10,(8,9))))))));
		ML	(18,(17,((16,(14,15)),(12,((4,(13,(0,(2,(1,3))))),((11,(9,10)),(5,(6,(7,8)))))))));
224	9,367	MP	(18,(17,((16,(14,15)),((5,(4,(2,(3,(1,0))))),(13,((10,(9,11)),(8,(12,(6,7)))))))));
		ML	(18,(17,((12,(16,(14,15))),(4,((13,(0,(1,3,2))),((10,(9,11)),(5,(6,(7,8)))))))));
225	9,840	MP	(18,(17,((16,(14,15)),((5,(4,(3,(0,(1,2))))),(13,(9,((6,(7,8)),(12,(10,11)))))))));
		ML	(18,((9,(10,11)),((12,(17,(16,(14,15)))),((5,(6,(7,8))),(4,(13,(0,(3,2,1))))))));
226	16,444	MP	(18,(13,(12,(((10,(8,9)),(7,(6,(4,5)))),((1,(2,(3,0))),(11,(17,(16,(14,15)))))))));
		ML	(18,(17,((16,(14,15)),(12,(4,((13,(0,(2,3,1))),((9,(10,11)),(5,(7,(6,8))))))))));
227	9,920	MP	(18,(14,((17,(15,16)),((5,(4,(0,(2,(1,3))))),(13,((12,(10,11)),(9,(8,(6,7)))))))));
		ML	(18,(17,((16,(14,15)),(((9,(10,11)),(8,(5,(6,7)))),(12,(4,(13,(0,(3,2,1)))))))));
228	1,2471	MP	(18,(17,((16,(14,15)),((5,(4,(3,(0,(1,2))))),(13,((10,(11,12)),(9,(6,(7,8)))))))));
		ML	(18,((17,(16,(14,15))),(12,((5,(7,(6,8))),((10,(9,11)),(4,(13,(0,(1,2,3)))))))));
229	1,7751	MP	(18,((17,(16,(14,15))),(((11,(9,10)),(7,(6,(4,5)))),(8,(13,(12,(1,(3,(2,0)))))))));
		MP	(18,(7,(((11,(9,10)),(17,(14,(12,13)))),((8,(4,(5,6))),(15,(16,(1,(3,(2,0)))))))));
		ML	(18,((4,(2,(0,(1,3)))),(13,((10,(9,11)),((5,(6,(7,8))),(12,(17,(16,(14,15)))))))));
230	7,613	MP	(18,((17,(16,(14,15))),(13,((12,(4,(3,(0,(1,2))))),((9,(10,11)),(5,(8,(6,7))))))));
		ML	(18,(17,((16,(14,15)),(((10,(9,11)),(5,(8,(6,7)))),(12,(4,(13,(2,(0,(1,3))))))))));
231	22,638	MP	(18,((16,(14,15)),(17,((4,(13,(2,(3,(1,0))))),((7,(5,6)),(12,(8,(11,(9,10)))))))));
		ML	(18,((17,(16,(14,15))),(12,((4,(13,(0,(2,(1,3))))),((9,(10,11)),(5,(7,(6,8))))))));
232	9,763	MP	(18,((17,(15,16)),(14,((5,(4,((2,3),(1,0)))),(6,((7,(8,9)),(10,(13,(11,12)))))))));
		ML	(18,(17,((16,(14,15)),(12,((4,(13,(0,(1,2,3)))),((9,(10,11)),(5,(6,(7,8)))))))));
233	3,923	MP	(18,(17,((16,(14,15)),((12,(4,(1,(3,(2,0))))),(13,((11,(9,10)),(5,(8,(6,7)))))))));
		MP	(18,(17,((16,(14,15)),(12,((13,(4,((1,3),(2,0)))),((11,(9,10)),(5,(6,(7,8)))))))));
		ML	(18,((17,(16,(14,15))),((4,(13,(0,2,3,1))),(12,((9,(10,11)),(5,(8,(6,7))))))));
234	14,445	MP	(18,(17,((16,(14,15)),(13,((11,(12,((2,3),(1,0)))),((8,(9,10)),(4,(6,(5,7)))))))));
		ML	(18,((12,(17,(16,(14,15)))),(4,(13,(0,(3,1,2)))),((9,(10,11)),(5,(8,(6,7))))));
235	6,589	MP	(18,(17,((12,(4,((2,3),(1,0)))),(13,((5,6),((16,(14,15)),((7,8),(11,(9,10)))))))));
		ML	(18,((5,(8,(6,7))),((10,(9,11)),((12,(17,(16,(14,15)))),(4,(13,(1,0,2,3)))))));
236	4,760	MP	(18,(17,((12,(13,14)),(6,(((15,16),(3,(1,(2,0)))),((4,(5,7)),(9,(10,(8,11)))))))));
		ML	(18,((17,(16,(14,15))),((10,(9,11)),((12,(5,(8,(6,7)))),(4,(13,(3,2,0,1)))))));
237	4,378	MP	(18,(16,((15,(13,14)),((4,(0,(3,(1,2)))),(11,(12,((9,(8,10)),((5,17),(6,7)))))))));
		ML	(18,(17,((16,(14,15)),(12,((5,(6,(7,8))),((9,(10,11)),(4,(13,(2,0,(1,3))))))))));
238	5,168	MP	(18,((17,(16,(14,15))),(5,(4,((13,(2,(3,(1,0)))),((10,(11,12)),(9,(6,(7,8)))))))));
		ML	(18,((17,(16,(14,15))),(12,((4,(13,(0,2,1,3))),((9,(10,11)),(5,(6,(7,8))))))));
239	4,004	MP	(18,((17,(16,(14,15))),(13,((5,(4,(0,(3,(1,2))))),((12,(10,11)),(9,(8,(6,7))))))));
		ML	(18,(12,((4,(13,(0,1,2,3))),((17,(16,(14,15))),((10,(9,11)),(5,(8,(6,7))))))));
240	6,971	MP	(18,((17,(16,(14,15))),((4,(2,(3,(1,0)))),(13,(12,((11,(9,10)),(8,(7,(5,6)))))))));
		ML	(18,(17,((16,(14,15)),((5,(7,(6,8))),(12,((11,(9,10)),(4,(13,(0,2,(1,3))))))))));

Table S1: Vibrionaceae 19–taxon large chromosome dataset LCBs and trees (continued).

LCB	Aligned bp	Algorithm	Tree topologies (Newick)
241	10,790	MP	(18,((16,(14,15)),((13,(11,((1,2),(3,0)))),((6,7),(5,(4,((12,17),(10,(8,9)))))))));
		ML	(18,(17,((16,(14,15)),(12,((5,(6,(7,8))),((10,(9,11)),(4,(13,(2,0,1,3)))))))));
242	3,407	MP	(18,((16,17),(8,(((9,(10,11)),(4,(7,(5,6)))),((12,(14,15)),(13,(0,(3,(1,2)))))))));
		ML	(18,(13,((0,2,1,3),(4,((8,(5,(6,7))),((9,(10,11)),((12,17),(16,(14,15)))))))));
243	7,533	MP	(18,(17,((16,(14,15)),((5,(4,(0,(3,(1,2))))),((13,(11,12)),(10,(6,(7,(8,9)))))))));
		MP	(18,((5,((1,2),(3,0))),((12,(10,11)),((9,(6,(7,8))),(17,(15,(16,(4,(13,14)))))))));
		ML	(18,((17,(16,(14,15))),(0,(1,2,3)),((10,(9,11)),((4,13),(12,(5,(7,(6,8))))))));
244	5,858	MP	(18,((16,17),((13,14),((11,(9,10)),((6,(5,(7,8))),((4,(12,15)),(0,(1,(2,3)))))))));
		ML	(18,((7,(8,(5,6))),((0,(2,1,3)),((13,(4,12)),((10,(9,11)),(17,(16,(14,15))))))));
245	11,791	MP	(18,((16,(14,15)),(13,(((10,(11,12)),(9,(8,(6,7)))),(5,((4,17),(1,(0,(2,3)))))))));
		ML	(18,(17,((16,(14,15)),(12,((4,(13,(3,2,0,1))),((9,(10,11)),(6,(5,(7,8)))))))));
246	2,714	MP	(18,(17,((5,((1,2),(3,0))),(13,((6,(10,(8,9))),((7,(11,12)),(4,(16,(14,15)))))))));
		ML	(18,(17,((16,(14,15)),(12,((4,(5,(8,(6,7)))),((11,(9,10)),(13,(0,(3,1,2)))))))));
247	5,800	MP	(18,((17,(15,16)),(14,((13,(12,(1,(3,(2,0))))),(8,((10,(9,11)),(4,(5,(6,7)))))))));
		ML	(18,((17,(16,(14,15))),((5,(7,(6,8))),((11,(9,10)),(12,(4,(13,(0,(3,2,1)))))))));
248	1,203	MP	(18,((4,(7,(5,6))),(8,((12,(10,11)),((0,(2,(1,3))),(13,((9,14),(17,(15,16)))))))));
		ML	(18,((5,(17,(16,(14,15)))),(12,((6,(7,8)),(13,(4,((10,(9,11)),(0,1,2,3))))))));
249	8,583	MP	(18,(17,(((12,(11,13)),(4,(6,(5,7)))),(9,((0,(1,(2,3))),(10,(8,(16,(14,15)))))))));
		ML	(18,((17,(16,(14,15))),((12,(8,(6,(5,7)))),(4,((10,(9,11)),(13,(0,1,3,2)))))));
250	1,447	MP	(18,((9,(7,(5,6))),((4,(0,(1,(2,3)))),(13,((10,(11,12)),(8,(17,(16,(14,15)))))))));
		ML	(18,((17,(16,(14,15))),((13,(0,3,1,2)),((4,12),((10,(9,11)),(8,(6,(5,7))))))));
251	23,433	MP	(18,(12,((10,(8,9)),((13,(17,(16,(14,15)))),((7,(4,(5,6))),(11,(0,(3,(1,2)))))))));
		ML	(18,((17,(16,(14,15))),((4,(13,(0,1,3,2))),(12,((9,(10,11)),(5,(6,(7,8))))))));
252	13,753	MP	(18,((0,(3,(1,2))),(12,((13,(11,(9,10))),((5,(7,(4,6))),(8,(17,(16,(14,15)))))))));
		ML	(18,((17,(16,(14,15))),((4,(13,(0,2,(1,3)))),(12,((10,(9,11)),(5,(8,(6,7))))))));
253	28,468	MP	(18,((17,(15,(13,14))),(12,((8,(1,(0,(2,3)))),(7,(16,((4,(5,6)),(11,(9,10)))))))));
		ML	(18,((4,(13,(1,0,2,3))),((10,(9,11)),((5,(6,(7,8))),(12,(17,(16,(14,15))))))));
254	8,722	MP	(18,((17,(16,(14,15))),(13,((12,(4,(0,(3,(1,2))))),((11,(9,10)),(8,(7,(5,6))))))));
		ML	(18,((0,1,2,3),(13,(4,((9,(10,11)),((7,(8,(5,6))),(12,(17,(16,(14,15))))))))));
255	19,319	MP	(18,((17,(16,(14,15))),(13,((12,(4,(0,(1,(2,3))))),((11,(9,10)),(5,(8,(6,7))))))));
		ML	(18,((17,(16,(14,15))),(12,((13,(4,(0,3,2,1))),((9,(10,11)),(8,(6,(5,7))))))));
256	4,293	MP	(18,((16,(14,15)),(17,((4,(13,(0,(2,(1,3))))),(12,((9,(10,11)),(5,(8,(6,7)))))))));
		ML	(18,((17,(16,(14,15))),(4,(13,(1,2,0,3))),(12,((9,(10,11)),(5,(8,(6,7)))))));
257	13,007	MP	(18,((16,(14,15)),(13,(((4,17),(0,(3,(1,2)))),(12,((11,(9,10)),(5,(8,(6,7)))))))));
		ML	(18,((17,(16,(14,15))),((4,(13,(0,1,2,3))),(12,((10,(9,11)),(5,(8,(6,7))))))));
258	9,871	MP	(18,((14,(17,(15,16))),((12,13),((4,(7,(5,6))),((10,(9,11)),(8,(3,(2,(1,0)))))))));
		ML	(18,(17,((16,(14,15)),((4,(13,(0,1,2,3))),(12,((9,(10,11)),(5,(8,(6,7)))))))));
259	3,647	MP	(18,((17,(0,(1,(2,3)))),(13,(8,((12,(16,(14,15))),((11,(9,10)),(7,(5,(4,6)))))))));
		MP	(18,(17,((16,(14,15)),((13,(4,(0,(2,(1,3))))),(12,((11,(9,10)),(5,(6,(7,8)))))))));

Table S1: Vibrionaceae 19–taxon large chromosome dataset LCBs and trees (continued).

LCB	Aligned bp	Algorithm	Tree topologies (Newick)
		ML	(18,(17,((16,(14,15)),((13,(0,(1,2,3))),(4,(12,((10,(9,11)),(5,(8,(6,7))))))))));
260	9,626	MP	(18,(17,((16,(14,15)),(12,((13,(4,((1,3),(2,0)))),((9,(10,11)),(8,(7,(5,6)))))))));
		ML	(18,(17,((16,(14,15)),(((9,(10,11)),(5,(7,(6,8)))),(12,(4,(13,(0,(3,1,2)))))))));
261	6,554	MP	(18,((16,17),(((1,2),(3,0)),((7,(5,6)),(9,((12,(10,11)),(13,((4,8),(14,15)))))))));
		ML	(18,(11,(10,9,((2,1,0,3),((4,13),(12,((5,(8,(6,7))),(17,(16,(14,15))))))))));
262	1,829	MP	(18,(13,((14,(17,(15,16))),(12,((4,(0,(1,(2,3)))),(5,((8,(6,7)),(11,(9,10)))))))));
		ML	(18,(12,((4,(13,(3,0,1,2))),((17,(16,(14,15))),((10,(9,11)),(5,(7,(6,8))))))));
263	22,464	MP	(18,((17,(13,14)),(16,(((9,15),(8,(6,7))),((10,(11,12)),(4,(5,((1,3),(2,0)))))))));
		ML	(18,((17,(16,(14,15))),((4,(13,(0,(2,(1,3))))),(12,((10,(9,11)),(8,(6,(5,7))))))));
264	14,861	MP	(18,((3,(0,(1,2))),((7,(5,6)),(13,(4,((17,(16,(14,15))),((8,12),(11,(9,10)))))))));
		ML	(18,((13,(3,0,2,1)),((4,(10,(9,11))),((5,(8,(6,7))),(12,(17,(16,(14,15))))))));
265	13,293	MP	(18,(13,((0,(3,(1,2))),(11,((12,(17,(16,(14,15)))),((8,(9,10)),(7,(4,(5,6)))))))));
		ML	(18,(17,((16,(14,15)),(12,(((5,8),(6,7)),((4,(10,(9,11))),(13,(1,3,0,2))))))));
266	2,467	MP	(18,((16,17),((9,(11,(10,12))),((6,(5,7)),((8,14),(4,((2,3),((13,15),(1,0)))))))));
		ML	(18,(17,((16,(14,15)),((4,(13,(0,2,1,3))),(12,((10,(9,11)),(8,(7,(5,6)))))))));
267	2,260	MP	(18,((16,(14,15)),(17,(13,((5,(4,((2,3),(1,0)))),((11,(10,12)),(9,(7,(6,8)))))))));
		ML	(18,(17,((16,(14,15)),((4,(13,(0,(3,2,1)))),((10,(9,11)),(12,(5,(8,(6,7)))))))));
268	4,118	MP	(18,((11,(10,(8,9))),(13,((12,(0,(3,(1,2)))),((16,(14,15)),(7,(17,(4,(5,6)))))))));
		ML	(18,(17,((16,(14,15)),(((11,(9,10)),(5,(8,(6,7)))),(12,(13,(4,(0,1,3,2))))))));
269	13,105	MP	(18,((17,(16,(14,15))),(13,((5,(4,(1,(2,(3,0))))),((12,(10,11)),(6,(9,(7,8))))))));
		ML	(18,(17,((16,(14,15)),((4,(2,(0,(1,3)))),((13,(9,(10,11))),(12,((5,6),(7,8))))))));
270	20,985	MP	(18,(17,((16,(14,15)),((5,(4,(2,(3,(1,0))))),(13,((12,(10,11)),(9,(6,(7,8)))))))));
		ML	(18,((17,(16,(14,15))),(12,((4,(13,(2,(0,(1,3))))),((10,(9,11)),(5,(8,(6,7))))))));
271	10,436	MP	(18,((14,(12,13)),(16,((15,(4,(0,(3,(1,2))))),(17,((11,(9,10)),(5,(8,(6,7)))))))));
		ML	(18,((17,(16,(14,15))),(12,((4,(13,((1,3),(0,2)))),((11,(9,10)),(8,(5,(6,7))))))));
272	3,882	MP	(18,((14,(12,13)),(16,((15,(4,(0,(3,(1,2))))),(17,((11,(9,10)),(5,(8,(6,7)))))))));
		MP	(18,(17,((16,(14,15)),((5,(4,(0,(2,(1,3))))),(13,((10,(11,12)),(9,(6,(7,8)))))))));
		MP	(18,(13,((5,(4,(0,(2,(1,3))))),((17,(16,(14,15))),((12,(10,11)),(9,(7,(6,8))))))));
		ML	(18,(15,(14,(16,(17,(12,((4,(13,(1,3,(0,2)))),((9,(10,11)),(5,(8,(6,7)))))))))));
273	6,231	MP	(18,((12,(0,(3,(1,2)))),((17,(13,(16,(14,15)))),(4,((11,(9,10)),(5,(6,(7,8))))))));
		ML	(18,((13,(2,0,1,3)),(4,((12,(17,(16,(14,15)))),((10,(9,11)),((5,6),(7,8)))))));
274	11,251	MP	(18,((5,(4,(0,(2,(1,3))))),((9,(6,(7,8))),(13,((10,(11,12)),(17,(16,(14,15))))))));
		ML	(18,((12,(16,(14,15))),(17,((4,(13,(1,0,2,3))),((9,(10,11)),(7,(8,(5,6))))))));
275	7,695	MP	(18,((17,(16,(14,15))),((4,(5,(0,(1,(2,3))))),(13,((10,(11,12)),(9,(7,(6,8))))))));
		MP	(18,((17,(16,(14,15))),((5,(4,((2,3),(1,0)))),(13,((12,(10,11)),(6,(9,(7,8))))))));
		ML	(18,(17,((12,(16,(14,15))),((4,(13,(0,3,2,1))),((10,(9,11)),(8,(5,(6,7))))))));
276	29,440	MP	(18,((17,(16,(14,15))),(13,((12,(4,((1,3),(2,0)))),((10,(9,11)),(5,(8,(6,7))))))));
		ML	(18,(17,((16,(14,15)),(12,((4,(13,(0,(3,1,2)))),((10,(9,11)),(5,(6,(7,8)))))))));
277	963	MP	(18,((13,(14,(15,(16,17)))),(((1,2),(3,0)),((5,11),(7,((12,(4,6)),(8,(9,10))))))));

Table S1: Vibrionaceae 19–taxon large chromosome dataset LCBs and trees (continued).

LCB	Aligned bp	Algorithm	Tree topologies (Newick)
		ML	(18,(17,((16,(14,15)),((4,(13,(0,3,1,2))),((5,(8,(6,7))),(12,(11,(9,10)))))))));
278	16,240	MP	(18,((9,(8,10)),(((4,11),(7,(5,6))),((17,(16,(14,15))),((12,13),(0,(3,(1,2))))))));
		MP	(18,((14,(17,(15,16))),((4,(1,(2,(3,0)))),((11,(9,10)),(5,((12,13),(8,(6,7))))))));
		ML	(18,(17,((16,(14,15)),(12,((4,(13,((1,3),(0,2)))),((10,(9,11)),(5,(7,(6,8)))))))));
279	9,934	MP	(18,((4,(8,(12,(14,(15,16))))),((2,(3,(1,0))),(17,(13,((5,(6,7)),(11,(9,10))))))));
		ML	(18,((17,(16,(14,15))),((4,(13,(0,(1,2,3)))),((10,(9,11)),(12,((5,6),(7,8)))))));
280	25,764	MP	(18,((17,(16,(14,15))),((13,(4,((1,2),(3,0)))),(12,((11,(9,10)),(5,(7,(6,8))))))));
		ML	(18,((12,(17,(16,(14,15)))),((13,(4,(0,(2,(1,3))))),((10,(9,11)),(8,(5,(6,7)))))));
281	6,358	MP	(18,((17,(16,(14,15))),((5,(4,(1,(0,(2,3))))),(13,((12,(10,11)),(9,(8,(6,7))))))));
		ML	(18,((16,(14,15)),(17,(12,((8,(5,(6,7))),((11,(9,10)),(4,(13,(2,(0,(1,3))))))))));
282	7,806	MP	(18,((5,(4,(0,(3,(1,2))))),((17,(16,(14,15))),(13,((10,(11,12)),(6,(9,(7,8))))))));
		ML	(18,((17,(16,(14,15))),(12,(((5,6),(7,8)),((10,(9,11)),(4,(13,(2,(0,1,3)))))))));
283	5,314	MP	(18,((17,(16,(14,15))),((5,(4,(3,(0,(1,2))))),((12,(10,11)),(13,(6,(7,(8,9))))))));
		MP	(18,((17,(16,(14,15))),((5,(4,(0,(1,(2,3))))),(13,((12,(10,11)),(9,(8,(6,7))))))));
		MP	(18,((13,(17,(16,(14,15)))),((5,(4,(3,(1,(2,0))))),((11,(10,12)),(6,(8,(7,9)))))));
		ML	(18,((16,(14,15)),(17,(4,((13,(2,(0,(1,3)))),((10,(9,11)),(12,(5,(8,(6,7))))))))));
284	5,126	MP	(18,(17,((16,(15,(13,14))),((12,(8,((1,2),(3,0)))),((9,(10,11)),(4,(5,(6,7))))))));
		ML	(18,(17,((16,(14,15)),(12,((13,(4,(0,(2,(1,3))))),((11,(9,10)),(5,(8,(6,7)))))))));
285	1,3804	MP	(18,((17,(16,(14,15))),(((12,(10,11)),(6,(7,(8,9)))),(13,(5,(4,((1,3),(2,0))))))));
		ML	(18,((16,(14,15)),(17,(12,((4,(13,(0,(2,3,1)))),((10,(9,11)),(8,(7,(5,6)))))))));
286	30,507	MP	(18,(5,((4,(0,(1,(2,3)))),((13,(14,(17,(15,16)))),((12,(10,11)),(6,(7,(8,9))))))));
		ML	(18,((4,(13,(0,(3,2,1)))),((12,(17,(16,(14,15)))),((9,(10,11)),(8,(5,(6,7)))))));
287	5,736	MP	(18,((9,(7,((6,8),(13,(10,(11,12)))))),((17,(16,(14,15))),(5,(4,(0,(1,(2,3))))))));
		ML	(18,((17,(16,(14,15))),(12,((8,(7,(5,6))),((11,(9,10)),(4,(13,((1,3),(0,2)))))))));
288	2,046	MP	(18,((17,(16,(14,15))),((5,(4,(0,(3,(1,2))))),(13,((12,(10,11)),(6,(8,(7,9))))))));
		ML	(18,(3,1,((0,2),(13,(4,((9,(10,11)),((5,(7,(6,8))),(17,(12,(16,(14,15))))))))));
289	1,152	MP	(18,((17,(16,(14,15))),((10,(13,(11,12))),((4,(7,(5,6))),(8,(9,((1,3),(2,0))))))));
		MP	(18,(5,((6,((13,(11,12)),(7,(10,(8,9))))),(17,((4,(16,(14,15))),(0,(1,(2,3))))))));
		MP	(18,(17,((6,(4,(11,((1,3),(2,0))))),((16,(14,15)),((5,(7,13)),(12,(10,(8,9))))))));
		ML	(18,((17,(16,(14,15))),(12,((4,(13,((1,3),(0,2)))),((11,(9,10)),(5,(7,(6,8))))))));
290	10,570	MP	(18,(17,((16,(14,15)),((9,(4,((2,3),(1,0)))),((8,(5,(6,7))),(13,(11,(10,12))))))));
		ML	(18,(17,((16,(14,15)),(12,((4,(13,((1,3),(0,2)))),((11,(9,10)),(5,(7,(6,8)))))))));
291	5,713	MP	(18,((16,(14,15)),(((10,(11,12)),(9,(7,(6,8)))),((13,17),(5,(4,((2,3),(1,0))))))));
		ML	(18,((17,(16,(14,15))),(12,((4,(13,(2,0,1,3))),((10,(9,11)),(5,(6,(7,8))))))));
292	7,861	MP	(18,(((8,(6,7)),(13,(12,(10,11)))),((9,(14,(17,(15,16)))),(5,(4,(0,(1,(2,3))))))));
		ML	(18,((12,(17,(16,(14,15)))),((4,(13,(0,1,2,3))),((9,(10,11)),((7,8),(5,6))))));
293	30,825	MP	(18,(11,((4,(5,(6,7))),((17,(10,(8,9))),((0,(2,(1,3))),((12,13),(16,(14,15))))))));
		ML	(18,((17,(16,(14,15))),((4,(13,(0,(2,(1,3))))),(12,((9,(10,11)),(5,(7,(6,8))))))));
294	8,398	MP	(18,(17,(((4,(7,8)),(12,(10,11))),((6,(3,(2,(1,0)))),(9,((5,13),(16,(14,15))))))));

Table S1: Vibrionaceae 19–taxon large chromosome dataset LCBs and trees (continued).

LCB	Aligned bp	Algorithm	Tree topologies (Newick)
		ML	(18,(17,((16,(14,15)),(12,((4,(13,(2,0,1,3))),((11,(9,10)),(5,(8,(6,7)))))))));
295	3,013	MP	(18,(((10,(8,9)),(12,(7,(5,(4,6))))),(17,(16,((11,(15,(13,14))),(0,(3,(1,2))))))));
		MP	(18,((14,(17,(15,16))),((5,(0,(2,(1,3)))),((10,(11,12)),(13,((4,6),(9,(7,8))))))));
		ML	(18,(6,(8,(7,(5,((10,(9,11)),((12,(16,(14,15))),(17,(4,(13,(3,1,0,2))))))))));
296	29,352	MP	(18,((17,(16,(14,15))),((5,(4,(0,(1,(2,3))))),(13,((12,(10,11)),(6,(7,(8,9))))))));
		ML	(18,(17,((16,(14,15)),((4,(13,(0,(1,3,2)))),(12,((11,(9,10)),(5,(6,(7,8)))))))));
297	945	MP	(18,((17,(16,(14,15))),((13,(4,(0,(3,(1,2))))),((11,(9,10)),(12,(5,(6,(7,8))))))));
		ML	(18,((16,(14,15)),(17,(12,((4,(13,(0,(2,(1,3))))),((10,(9,11)),(8,(6,(5,7)))))))));
298	1,916	MP	(18,((5,(4,((13,(11,12)),(10,(9,(7,8)))))),(17,((16,(14,15)),(6,(3,(1,(2,0))))))));
		MP	(18,((17,(16,(14,15))),((5,(4,(0,(1,(2,3))))),((6,(7,8)),(13,(12,(11,(9,10))))))));
		ML	(18,((12,(17,(16,(14,15)))),((4,(13,(0,3,1,2))),((10,(9,11)),(5,(7,(6,8)))))));
299	24,454	MP	(18,((17,(16,(14,15))),(13,((5,(4,((2,3),(1,0)))),((11,(10,12)),(6,(7,(8,9))))))));
		ML	(18,((17,(16,(14,15))),((4,(13,(0,(2,(1,3))))),((9,(10,11)),(12,(5,(8,(6,7))))))));
300	14,405	MP	(18,((17,(16,(14,15))),((5,(4,(1,(2,(3,0))))),(13,((11,(10,12)),(7,(9,(6,8))))))));
		ML	(18,((17,(16,(14,15))),((13,(4,(0,(1,2,3)))),(12,((9,(10,11)),(5,(6,(7,8))))))));
301	7,980	MP	(18,((17,(16,(14,15))),((4,(5,(0,(3,(1,2))))),((10,(11,12)),(13,(9,(7,(6,8))))))));
		ML	(18,((13,(4,(0,(2,(1,3))))),((9,(10,11)),((5,(7,(6,8))),(12,(17,(16,(14,15))))))));
302	4,579	MP	(18,((13,(6,(9,(7,8)))),((4,(0,(2,(1,3)))),((11,(10,12)),(5,(17,(16,(14,15))))))));
		ML	(18,(17,((16,(14,15)),(12,((4,(13,(0,3,2,1))),((10,(9,11)),(5,(6,(7,8)))))))));
303	26,356	MP	(18,(((13,(12,(10,11))),(17,(16,(14,15)))),((5,(9,(8,(6,7)))),(4,(0,(3,(1,2)))))));
		ML	(18,((12,(17,(16,(14,15)))),((4,(13,(3,1,(0,2)))),((9,(10,11)),(5,(6,(7,8)))))));
304	36,797	MP	(18,((17,(4,(0,(2,(1,3))))),((14,((12,13),(15,16))),((11,(9,10)),(7,(5,(6,8)))))));
		ML	(18,((17,(16,(14,15))),((13,(4,(3,(0,1,2)))),(12,((10,(9,11)),(8,(6,(5,7))))))));
305	9,437	MP	(18,(((16,(14,15)),(17,(2,(1,(3,0))))),((12,(4,13)),((11,(9,10)),(8,(7,(5,6)))))));
		MP	(18,((11,(9,10)),((13,(0,(3,(1,2)))),((4,(7,(5,6))),((12,(8,14)),(17,(15,16)))))));
		ML	(18,((4,(13,(2,1,0,3))),((11,(9,10)),((8,(7,(5,6))),(12,(17,(16,(14,15))))))));
306	19,956	MP	(18,((17,(16,(14,15))),(((9,(10,11)),(5,(8,(6,7)))),((4,(12,13)),(0,(1,(2,3)))))));
		ML	(18,(17,((16,(14,15)),(((9,(10,11)),(5,(8,(6,7)))),(12,(4,(13,(0,(2,1,3)))))))));
