# Supplementary material for: Genome-level homology and phylogeny of Vibrionaceae (Gammaproteobacteria: Vibrionales) with three new complete genome sequences
Source: BMC Microbiol. 2013 Apr 11;13:80. doi: 10.1186/1471-2180-13-80 (PMC3663674; doi:10.1186/1471-2180-13-80)
Supplement: Additional file 2 — Table S2. Vibrionaceae 19–Taxon Small Chromosome Dataset LCBs and Trees. [file 1471-2180-13-80-S2.pdf]

Table S2: Vibrionaceae 19–taxon small chromosome dataset LCBs and trees.

LCB	Aligned bp	Algorithm	Tree topologies (Newick)
1	6,886	MP	(18,(10,((9,(11,(4,5))),((17,(15,16)),((0,(2,(1,3))),((6,(7,8)),(14,(12,13))))))));
		ML	(18,((16,(14,15)),(17,(12,((5,(8,(6,7))),((10,(9,11)),(4,(13,(0,(1,2,3))))))))));
2	12,859	MP	(18,(16,((17,(15,(13,14))),((12,(10,(9,11))),((8,(6,7)),(5,(4,(0,(2,(1,3))))))))));
		ML	(18,(12,((4,(13,(0,(1,2,3)))),((9,(10,11)),((17,(16,(14,15))),(5,(7,(6,8))))))));
3	3,317	MP	(18,(8,((11,(9,10)),(16,((0,(2,(1,3))),(17,((14,(13,15)),(7,(12,(6,(4,5))))))))))));
		ML	(18,((11,(9,10)),((4,13),(0,1,2,3)),(12,((17,(16,(14,15))),(8,(5,(6,7)))))));
4	3,675	MP	(18,((8,(14,((7,(5,6)),(13,(11,(10,12)))))),((3,(2,(0,1))),(9,(4,(17,(15,16)))))));
		ML	(18,((17,(16,(14,15))),((0,1,2,3),((4,13),((11,(9,10)),(12,(5,(8,(6,7)))))))));
5	15,492	MP	(18,((0,(3,(1,2))),(4,(5,((6,(9,(7,8))),((13,(11,12)),(10,(17,(16,(14,15))))))))));
		ML	(18,((17,(16,(14,15))),((4,(13,(0,(2,(1,3))))),((11,(9,10)),(12,(5,(8,(6,7))))))));
6	4,483	MP	(18,(15,(8,(((1,3),(0,2)),((6,(16,(13,14))),(9,(17,((4,(5,7)),(10,(11,12))))))))));
		ML	(18,(16,((14,15),((17,12),((10,(9,11)),(13,((0,1,2,3),(4,(8,(6,(5,7)))))))))));
7	8,457	MP	(18,(11,((14,(12,13)),(16,((0,(1,(2,3))),(17,(6,((4,(5,7)),(15,(10,(8,9)))))))))));
		ML	(18,(17,((16,(14,15)),(((4,13),(0,1,2,3)),(12,((10,(9,11)),(5,(7,(6,8)))))))));
8	8,852	MP	(18,((17,(15,16)),((6,(5,(4,(0,(2,(1,3)))))),(14,((9,(7,8)),(12,(10,(11,13))))))));
		ML	(18,((16,(14,15)),(17,((13,(4,(0,1,2,3))),(12,((11,(9,10)),(5,(7,(6,8)))))))));
9	9,283	MP	(18,(14,((17,(15,16)),((5,(4,(0,(3,(1,2))))),(13,((10,(11,12)),(6,(8,(7,9)))))))));
		ML	(18,((17,(16,(14,15))),((4,(13,(0,(2,(1,3))))),(12,((9,(10,11)),(5,(7,(6,8))))))));
10	6,334	MP	(18,((11,(9,10)),((15,(16,17)),(8,((6,7),(5,(12,(13,(4,(14,(3,(1,(0,2)))))))))))));
		ML	(18,(17,((16,(14,15)),(4,(12,(((5,8),(6,7)),((9,(10,11)),(13,(1,(0,2,3))))))))));
11	4,517	MP	(18,((15,(16,17)),((7,(6,8)),((4,((12,13),(11,(9,10)))),((5,14),(0,(2,(1,3))))))));
		MP	(18,((15,(16,17)),((8,(6,7)),((4,((12,13),(11,(9,10)))),((5,14),(0,(2,(1,3))))))));
		ML	(18,((17,(16,(14,15))),(12,(((4,13),(0,(1,2,3))),((10,(9,11)),(5,(6,(7,8))))))));
12	3,533	MP	(18,((15,16),(17,(11,((14,(12,13)),((5,(6,(4,7))),((8,9),(10,(0,(2,(1,3)))))))))));
		ML	(18,((16,(14,15)),(17,((0,1,2,3),((12,(4,13)),((10,(9,11)),(5,(8,(6,7)))))))));
13	6,938	MP	(18,((16,17),((6,7),(4,(11,(12,((15,(13,14)),((8,(9,10)),(5,(0,(3,(1,2)))))))))));
		ML	(18,((16,(14,15)),(17,(12,((4,(13,(0,(2,(1,3))))),((9,(10,11)),(5,(7,(6,8)))))))));
14	9,316	MP	(18,(16,((15,(13,14)),(17,(5,(((1,3),(0,2)),((7,(6,8)),(4,(12,(9,(10,11)))))))))));
		ML	(18,((16,(14,15)),(17,(12,(((4,13),(0,1,2,3)),((10,(9,11)),(5,(8,(6,7)))))))));
15	18,184	MP	(18,(((12,(11,13)),(6,(8,(9,10)))),((17,(15,16)),(14,((4,7),(5,(0,(3,(1,2)))))))));
		MP	(18,(((13,(11,12)),(6,(8,(9,10)))),((17,(15,16)),(14,((4,7),(5,(0,(3,(1,2)))))))));
		ML	(18,(17,((16,(14,15)),(((4,13),(0,(1,2,3))),((5,(6,(7,8))),(12,(11,(9,10))))))));
16	29,591	MP	(18,(11,((9,(7,8)),((6,(4,5)),(((2,3),(0,1)),((10,12),(13,(14,(17,(15,16))))))))));
		ML	(18,((5,7),(6,((8,(17,(16,(15,14)))),((9,(10,11)),(12,((4,13),(0,2,(1,3)))))))));
17	4,910	MP	(18,((16,17),(14,(12,(13,(((2,3),(0,1)),(4,(15,((11,(9,10)),(8,(5,(6,7)))))))))));
		ML	(18,(((10,(9,11)),(5,(7,(6,8)))),((17,(16,(14,15))),(12,(4,(13,((1,3),(0,2))))))));
18	7,176	MP	(18,(17,(4,(((1,3),(0,2)),(8,((5,(6,7)),(13,((16,(14,15)),(9,(10,(11,12)))))))))));
		ML	(18,((13,((4,17),(0,1,2,3))),((9,(10,11)),(((5,6),(7,8)),(12,(16,(14,15)))))));
19	8,079	MP	(18,(17,(15,((14,(12,13)),(16,(11,((4,(0,(2,(1,3)))),((7,(5,6)),(8,(9,10)))))))))));

Table S2: Vibrionaceae 19–taxon small chromosome dataset LCBs and trees (continued).

LCB	Aligned bp	Algorithm	Tree topologies (Newick)
		ML	(18,(17,((16,(14,15)),(12,((4,(13,(0,(1,2,3)))),((10,(9,11)),(8,(7,(5,6)))))))));
20	10,026	MP	(18,(11,((8,9),(17,(12,(10,(13,(5,(7,(4,(6,((14,(15,16)),(0,(2,(1,3)))))))))))))));
		MP	(18,(11,((8,9),(17,(12,(10,(13,(5,(7,(4,(6,((16,(14,15)),(0,(2,(1,3)))))))))))))));
		ML	(18,((10,(9,11)),((5,(6,(7,8))),(12,((4,(17,(16,(14,15)))),(13,(0,1,2,3)))))));
21	5,655	MP	(18,(14,((0,(2,(1,3))),(7,(12,((17,(15,16)),(13,((4,(5,6)),(8,(11,(9,10)))))))))));
		ML	(18,(17,((16,(14,15)),((4,(13,(0,(1,2,3)))),(12,((9,(10,11)),(5,(8,(6,7)))))))));
22	7,884	MP	(18,(14,(5,(((7,(6,8)),(13,(9,(12,(10,11))))),(4,((17,(15,16)),(0,(2,(1,3)))))))));
		ML	(18,(6,7,(8,(5,(12,((17,(16,(14,15))),(4,(13,((9,(10,11)),(0,(1,2,3)))))))))));
23	12,137	MP	(18,((10,(13,((7,(5,6)),(9,(11,12))))),((17,(16,(14,15))),(4,(8,(0,(2,(1,3))))))));
		ML	(18,((17,(16,(14,15))),((12,(7,(6,(5,8)))),(13,((4,(10,(9,11))),(0,1,2,3))))));
24	4,709	MP	(18,(14,((17,(15,16)),(12,((13,(11,((1,3),(0,2)))),((10,(8,9)),(4,(7,(5,6)))))))));
		ML	(18,((17,(16,(14,15))),(((9,(10,11)),(5,(8,(6,7)))),(12,(4,(13,(0,1,2,3)))))));
25	8,571	MP	(18,((16,17),(((4,10),(14,15)),(5,((6,(0,(2,(1,3)))),((7,(8,9)),(11,(12,13))))))));
		ML	(18,(17,((16,(14,15)),(12,(((4,13),(0,(1,2,3))),((9,(10,11)),(5,(8,(6,7)))))))));
26	14,483	MP	(18,(17,((9,(8,(6,7))),(4,((0,(2,(1,3))),((13,(11,12)),(5,(10,(16,(14,15))))))))));
		ML	(18,(((9,(10,11)),((5,6),(7,8))),(12,((17,(16,(14,15))),(4,(13,(0,(1,2,3))))))));
27	3,394	MP	(18,((17,(16,(14,15))),(13,(11,((8,(9,10)),((5,(6,7)),(12,(4,(0,(2,(1,3)))))))))));
		MP	(18,((17,(16,(14,15))),(13,((8,(9,10)),(11,((5,(6,7)),(12,(4,(0,(2,(1,3)))))))))));
		ML	(18,(11,10,((9,17),((4,(13,(0,(1,2,3)))),(8,((5,(6,7)),(12,(16,(14,15)))))))));
28	12,941	MP	(18,(5,(4,(17,(12,(9,((7,8),(6,((13,(14,15)),((16,(10,11)),(3,(2,(0,1)))))))))))));
		ML	(18,((13,(4,(0,(1,2,3)))),(17,((12,(16,(14,15))),(8,(6,(7,(5,(9,(10,11))))))))));
29	12,274	MP	(18,((16,(14,15)),((0,(2,(1,3))),((5,(6,7)),(13,(9,(11,(10,(12,(8,(4,17)))))))))));
		MP	(18,((16,(14,15)),((0,(2,(1,3))),((5,(6,7)),(13,((9,11),(10,(12,(8,(4,17))))))))));
		ML	(18,((16,(17,(14,15))),(5,(8,(7,(6,((4,(9,(10,11))),(12,(13,(0,(1,2,3)))))))))));
30	6,769	MP	(18,(14,((17,(15,16)),((5,(4,(0,(2,(1,3))))),((9,(6,(7,8))),(13,(10,(11,12))))))));
		ML	(18,(17,((16,(14,15)),(((9,(10,11)),(5,(8,(6,7)))),(12,(4,(13,(0,(1,2,3)))))))));
31	8,239	MP	(18,((4,5),((11,(10,12)),(9,((8,(6,7)),(13,((17,(15,16)),(0,(14,(2,(1,3)))))))))));
		ML	(18,((13,(0,(1,2,3))),(4,((12,(16,(14,15))),((5,(6,(7,8))),(17,(9,(10,11))))))));
32	7,550	MP	(18,(17,((16,(14,15)),(11,((10,(8,9)),(13,(4,((12,(7,(5,6))),(3,(1,(0,2)))))))))));
		ML	(18,(17,(12,(4,(13,(2,(0,1,3))))),((5,(8,(6,7))),((16,(14,15)),(11,(9,10))))));
33	10,552	MP	(18,(17,(14,((15,16),((10,(4,5)),((3,(1,(0,2))),(9,((8,(6,7)),(13,(11,12)))))))))));
		ML	(18,((17,(16,(14,15))),(13,((2,(0,1,3)),(4,(12,((11,(9,10)),(8,(7,(5,6))))))))));
34	11,554	MP	(18,((3,(1,(0,2))),(13,(4,((17,(16,(14,15))),(12,((10,(9,11)),(8,(6,(5,7))))))))));
		ML	(18,((10,(9,11)),(12,(8,(5,(6,7)))),((17,(16,(14,15))),(4,(13,(0,1,2,3))))));
35	11,718	MP	(18,(4,(5,((10,((2,3),(0,1))),((16,(14,15)),((6,(8,(7,9))),(11,(13,(12,17)))))))));
		ML	(18,(((9,(10,11)),(6,(7,(5,8)))),(17,((4,(12,(16,(14,15)))),(13,(1,3,(0,2)))))));
36	16,953	MP	(18,((16,17),((14,15),(13,((7,(6,8)),((10,(11,12)),(9,((4,5),((2,3),(0,1))))))))));
		ML	(18,((16,(14,15)),(((9,(10,11)),(8,(6,(5,7)))),(17,((4,12),(13,((1,3),(0,2))))))));
37	86,301	MP	(18,((16,(14,15)),(17,(((11,(12,13)),(10,(7,(8,9)))),(6,(5,(4,((2,3),(0,1)))))))));

Table S2: Vibrionaceae 19–taxon small chromosome dataset LCBs and trees (continued).

LCB	Aligned bp	Algorithm	Tree topologies (Newick)
		ML	(18,(17,((16,(14,15)),(((10,(9,11)),(5,(8,(6,7)))),(12,(4,(13,((1,3),(0,2)))))))));
