# Supplementary material for: Genome-level homology and phylogeny of Vibrionaceae (Gammaproteobacteria: Vibrionales) with three new complete genome sequences
Source: BMC Microbiol. 2013 Apr 11;13:80. doi: 10.1186/1471-2180-13-80 (PMC3663674; doi:10.1186/1471-2180-13-80)
Supplement: Additional file 3 — Table S3. Genes Found in RAST Subsystems for All Species Part 1. [file 1471-2180-13-80-S3.pdf]

Table S3: Genes Found in RAST Subsystems for All Species Part 1:

(A = *P. angustum*, B = *P. damselae*, C = *A. fischeri* ES114, D = *V. gazogenes*, E = *A. logei*, F = *P. profundum*, G = *A. salmonicida*, H = *P.* sp. SKA34, I = *S. oneidensis*, J = *V. alginolyticus* 12, K = *V. anguillarum*).

| RAST Subsystem | # in Fig. 7 | A | B | C | D | E | F | G | H | I | J | K |
|---|---|---|---|---|---|---|---|---|---|---|---|---|
| Cofactors, Vitamins, Prosthetic Groups, Pigments | 1 | 244 | 301 | 287 | 354 | 193 | 336 | 277 | 190 | 307 | 252 | 277 |
| Cell Wall and Capsule | 2 | 122 | 180 | 213 | 71 | 111 | 184 | 247 | 106 | 147 | 134 | 195 |
| Virulence, Disease and Defense | 3 | 99 | 89 | 88 | 94 | 100 | 113 | 85 | 82 | 86 | 87 | 66 |
| Potassium Metabolism | 4 | 35 | 31 | 28 | 39 | 26 | 30 | 30 | 27 | 30 | 36 | 20 |
| Photosynthesis | | 0 | 0 | 0 | 0 | 0 | 0 | 0 | 0 | 0 | 0 | 0 |
| Miscellaneous | 5 | 200 | 160 | 222 | 180 | 168 | 171 | 214 | 170 | 228 | 165 | 135 |
| Phages, Prophages, Transposable elements, Plasmids | 6 | 3 | 10 | 23 | 11 | 36 | 11 | 24 | 3 | 26 | 7 | 5 |
| Membrane Transport | 7 | 177 | 215 | 195 | 150 | 231 | 191 | 291 | 117 | 144 | 185 | 153 |
| Iron acquisition and metabolism | 8 | 75 | 38 | 70 | 11 | 94 | 49 | 75 | 84 | 47 | 69 | 25 |
| RNA Metabolism | 9 | 209 | 209 | 286 | 189 | 114 | 234 | 230 | 143 | 268 | 187 | 198 |
| Nucleosides and Nucleotides | 10 | 90 | 116 | 110 | 115 | 70 | 162 | 105 | 100 | 100 | 107 | 108 |
| Protein Metabolism | 11 | 233 | 265 | 253 | 171 | 206 | 198 | 247 | 191 | 262 | 139 | 212 |
| Cell Division and Cell Cycle | 12 | 43 | 38 | 44 | 40 | 48 | 32 | 48 | 34 | 36 | 23 | 32 |
| Motility and Chemotaxis | 13 | 149 | 93 | 115 | 37 | 120 | 60 | 109 | 148 | 123 | 163 | 138 |
| Regulation and Cell signaling | 14 | 98 | 90 | 108 | 114 | 108 | 110 | 107 | 82 | 66 | 107 | 92 |
| Secondary Metabolism | | 0 | 0 | 0 | 0 | 0 | 2 | 0 | 0 | 0 | 0 | 3 |
| DNA Metabolism | 15 | 148 | 154 | 136 | 123 | 138 | 110 | 131 | 129 | 146 | 133 | 148 |
| Fatty Acids, Lipids and Isoprenoids | 16 | 133 | 116 | 114 | 160 | 90 | 158 | 111 | 125 | 117 | 110 | 112 |
| Nitrogen Metabolism | 17 | 37 | 35 | 39 | 6 | 7 | 83 | 40 | 30 | 41 | 49 | 27 |
| Dormancy and Sporulation | 18 | 7 | 4 | 6 | 2 | 11 | 2 | 4 | 7 | 6 | 3 | 8 |
| Respiration | 19 | 194 | 152 | 109 | 132 | 172 | 209 | 115 | 168 | 194 | 147 | 96 |
| Stress Response | 20 | 170 | 152 | 135 | 190 | 146 | 172 | 119 | 152 | 124 | 179 | 119 |
| Metabolism of Aromatic Compounds | 21 | 10 | 3 | 3 | 11 | 6 | 12 | 6 | 13 | 12 | 10 | 11 |
| Amino Acids and Derivatives | 22 | 364 | 357 | 338 | 486 | 335 | 492 | 335 | 316 | 384 | 459 | 350 |
| Sulfur Metabolism | 23 | 33 | 28 | 28 | 54 | 35 | 32 | 22 | 28 | 29 | 17 | 28 |
| Phosphorus Metabolism | 24 | 62 | 41 | 35 | 64 | 38 | 50 | 40 | 48 | 46 | 46 | 41 |
| Carbohydrates | 25 | 392 | 413 | 355 | 745 | 361 | 610 | 332 | 322 | 268 | 414 | 414 |
| RNAs | 26 | 130 | 223 | 156 | 121 | 125 | 161 | 141 | 148 | 129 | 70 | 108 |
| coding sequences | | 4403 | 4408 | 3766 | 5616 | 4807 | 5358 | 4259 | 4175 | 4486 | 4536 | 3687 |
