# Supplementary material for: Genome-level homology and phylogeny of Vibrionaceae (Gammaproteobacteria: Vibrionales) with three new complete genome sequences
Source: BMC Microbiol. 2013 Apr 11;13:80. doi: 10.1186/1471-2180-13-80 (PMC3663674; doi:10.1186/1471-2180-13-80)
Supplement: Additional file 4 — Table S6. Vibrionaceae 19–Taxon Random Subset Datasets LCBs and Trees. [file 1471-2180-13-80-S4.pdf]

Table S6: Vibrionaceae 19–taxon random subset datasets LCBs and trees.

Chromosome	Aligned bp	Algorithm	Tree topologies (Newick)
Large	10,000	MP	(18,(17,((16,(14,15)),((13,(4,(0,(2,(1,3))))),(12,((10,(9,11)),(5,(8,(6,7)))))))));
		ML	(18,((17,(16,(14,15))),(12,((4,(13,(0,(2,(1,3))))),((10,(9,11)),(5,(8,(6,7))))))));
	20,000	MP	(18,((17,(16,(14,15))),((4,(13,(0,(2,(1,3))))),(12,((10,(9,11)),(8,(5,(6,7))))))));
		ML	(18,((17,(16,(14,15))),(12,((4,(13,(0,(2,(1,3))))),((9,(10,11)),(5,(8,(6,7))))))));
	30,000	MP	(18,((17,(16,(14,15))),((4,(13,(0,(2,(1,3))))),(12,((10,(9,11)),(5,(8,(6,7))))))));
		ML	(18,((17,(16,(14,15))),(12,((4,(13,(0,(2,(1,3))))),((9,(10,11)),(5,(6,(7,8))))))));
	40,000	MP	(18,((17,(16,(14,15))),((13,(4,(0,(2,(1,3))))),(12,((10,(9,11)),(5,(8,(6,7))))))));
		ML	(18,((17,(16,(14,15))),((4,(13,(0,(2,(1,3))))),(12,((9,(10,11)),(5,(8,(6,7))))))));
	50,000	MP	(18,((17,(16,(14,15))),((4,(13,(0,(2,(1,3))))),(12,((10,(9,11)),(5,(8,(6,7))))))));
		ML	(18,((17,(16,(14,15))),((4,(13,(0,(2,(1,3))))),(12,((10,(9,11)),(5,(8,(6,7))))))));
	100,000	MP	(18,((17,(16,(14,15))),((13,(4,(0,(2,(1,3))))),(12,((10,(9,11)),(5,(8,(6,7))))))));
		ML	(18,(17,((16,(14,15)),(12,((4,(13,(0,(2,(1,3))))),((11,(9,10)),(5,(8,(6,7)))))))));
	200,000	MP	(18,(17,((16,(14,15)),((4,(13,(0,(2,(1,3))))),(12,((10,(9,11)),(5,(8,(6,7)))))))));
		ML	(18,((17,(16,(14,15))),(12,((4,(13,(0,(2,(1,3))))),((10,(9,11)),(5,(8,(6,7))))))));
	300,000	MP	(18,(17,((16,(14,15)),((4,(13,(0,(2,(1,3))))),(12,((10,(9,11)),(5,(8,(6,7)))))))));
		ML	(18,((17,(16,(14,15))),(12,((4,(13,(0,(2,(1,3))))),((10,(9,11)),(5,(8,(6,7))))))));
	400,000	MP	(18,(17,((16,(14,15)),((4,(13,(0,(2,(1,3))))),(12,((10,(9,11)),(5,(8,(6,7)))))))));
		ML	(18,((17,(16,(14,15))),(12,((4,(13,(0,(2,(1,3))))),((11,(9,10)),(5,(8,(6,7))))))));
	500,000	MP	(18,((17,(16,(14,15))),((4,(13,(0,(2,(1,3))))),(12,((10,(9,11)),(5,(8,(6,7))))))));
		ML	(18,((17,(16,(14,15))),(12,((4,(13,(0,(2,(1,3))))),((10,(9,11)),(5,(8,(6,7))))))));
	1,000,000	MP	(18,(17,((16,(14,15)),((4,(13,(0,(2,(1,3))))),(12,((10,(9,11)),(5,(8,(6,7)))))))));
		ML	(18,(17,((16,(14,15)),((4,(13,(0,(2,(1,3))))),(12,((10,(9,11)),(5,(8,(6,7)))))))));
Small	10,000	MP	(18,((16,(14,15)),(17,((4,(13,(0,(2,(1,3))))),(12,((9,(10,11)),(8,(5,(6,7)))))))));
		ML	(18,((17,(16,(14,15))),(12,((4,(13,(1,3,(2,0)))),((11,(10,9)),(5,(8,(6,7))))))));
	20,000	MP	(18,((16,(14,15)),(17,((4,(13,(0,(2,(1,3))))),(12,((9,(10,11)),(8,(5,(6,7)))))))));
		ML	(18,((16,(14,15)),(17,((4,(13,(0,(2,(1,3))))),(12,((9,(10,11)),(8,(5,(6,7)))))))));
	30,000	MP	(18,((16,(14,15)),(17,((4,(13,(0,(2,(1,3))))),(12,((9,(10,11)),(8,(5,(6,7)))))))));
		ML	(18,((16,(14,15)),(17,((4,(13,(0,(2,(1,3))))),(12,((9,(10,11)),(8,(5,(6,7)))))))));
	40,000	MP	(18,((16,(14,15)),(17,((4,(13,(0,(2,(1,3))))),(12,((9,(10,11)),(8,(5,(6,7)))))))));
		ML	(18,((17,(16,(14,15))),(12,((4,(13,(0,(2,(1,3))))),((10,(11,9)),(5,(8,(6,7))))))));
	50,000	MP	(18,((16,(14,15)),(17,((4,(13,(0,(2,(1,3))))),(12,((9,(10,11)),(8,(5,(6,7)))))))));
		ML	(18,((17,(16,(14,15))),((4,(13,(0,(2,(1,3))))),(12,((9,(10,11)),(5,(8,(6,7))))))));
	100,000	MP	(18,((16,(14,15)),(17,((4,(13,(0,(2,(1,3))))),(12,((9,(10,11)),(8,(5,(6,7)))))))));
		ML	(18,((17,(16,(14,15))),(12,((4,(13,(0,(2,(1,3))))),((9,(10,11)),(5,(8,(6,7))))))));
	200,000	MP	(18,((16,(14,15)),(17,((4,(13,(0,(2,(1,3))))),(12,((9,(10,11)),(8,(5,(6,7)))))))));

Table S6: Vibrionaceae 19–taxon random subset datasets LCBs and trees (continued).

Chromosome	Aligned bp	Algorithm	Tree topologies (Newick)
		ML	(18,((17,(16,(14,15))),(12,((4,(13,(0,(2,(1,3))))),((9,(10,11)),(5,(8,(6,7))))))));
	300,000	MP	(18,((16,(14,15)),(17,((4,(13,(0,(2,(1,3))))),(12,((9,(10,11)),(8,(5,(6,7)))))))));
		ML	(18,((17,(16,(14,15))),(12,((4,(13,(0,(2,(1,3))))),((9,(10,11)),(5,(8,(6,7))))))));
both	10,000	MP	(18,(17,((16,(14,15)),((4,(13,(0,(2,(1,3))))),(12,((10,(9,11)),(8,(5,(6,7)))))))));
		ML	(18,(17,((16,(14,15)),((4,(13,(0,(2,(1,3))))),(12,((10,(9,11)),(5,(8,(6,7)))))))));
	20,000	MP	(18,(17,((16,(14,15)),((4,(13,(0,(2,(1,3))))),(12,((10,(9,11)),(5,(8,(6,7)))))))));
		ML	(18,((17,(16,(14,15))),(12,((4,(13,(0,(2,(1,3))))),((11,(9,10)),(5,(6,(7,8))))))));
	30,000	MP	(18,(17,((16,(14,15)),((4,(13,(0,(2,(1,3))))),(12,((10,(9,11)),(5,(8,(6,7)))))))));
		ML	(18,((17,(16,(14,15))),(12,((4,(13,(0,(2,(1,3))))),((9,(10,11)),(5,(8,(6,7))))))));
	40,000	MP	(18,((17,(16,(14,15))),((4,(13,(0,(2,(1,3))))),(12,((10,(9,11)),(5,(8,(6,7))))))));
		ML	(18,((17,(16,(14,15))),(12,((4,(13,(0,(2,(1,3))))),((10,(9,11)),(5,(8,(6,7))))))));
	50,000	MP	(18,(17,((16,(14,15)),((4,(13,(0,(2,(1,3))))),(12,((10,(9,11)),(5,(8,(6,7)))))))));
		ML	(18,((17,(16,(14,15))),(12,((4,(13,(0,(2,(1,3))))),((9,(10,11)),(5,(8,(6,7))))))));
	100,000	MP	(18,(17,((16,(14,15)),((4,(13,(0,(2,(1,3))))),(12,((10,(9,11)),(8,(5,(6,7)))))))));
		ML	(18,((17,(16,(14,15))),(12,((4,(13,(0,(2,(1,3))))),((10,(9,11)),(5,(8,(6,7))))))));
	200,000	MP	(18,(17,((16,(14,15)),((4,(13,(0,(2,(1,3))))),(12,((10,(9,11)),(5,(8,(6,7)))))))));
		ML	(18,((17,(16,(14,15))),(12,((4,(13,(0,(2,(1,3))))),((9,(10,11)),(5,(8,(6,7))))))));
	300,000	MP	(18,(17,((16,(14,15)),((4,(13,(0,(2,(1,3))))),(12,((10,(9,11)),(5,(8,(6,7)))))))));
		ML	(18,((17,(16,(14,15))),(12,((4,(13,(0,(2,(1,3))))),((9,(10,11)),(5,(8,(6,7))))))));
	400,000	MP	(18,(17,((16,(14,15)),((4,(13,(0,(2,(1,3))))),(12,((10,(9,11)),(5,(8,(6,7)))))))));
		ML	(18,((17,(16,(14,15))),(12,((4,(13,(0,(2,(1,3))))),((9,(10,11)),(5,(8,(6,7))))))));
	500,000	MP	(18,(17,((16,(14,15)),((4,(13,(0,(2,(1,3))))),(12,((10,(9,11)),(5,(8,(6,7)))))))));
		ML	(18,((17,(16,(14,15))),(12,((4,(13,(0,(2,(1,3))))),((9,(10,11)),(5,(8,(6,7))))))));
	1,000,000	MP	(18,(17,((16,(14,15)),((4,(13,(0,(2,(1,3))))),(12,((10,(9,11)),(5,(8,(6,7)))))))));
		ML	(18,(17,((16,(14,15)),((4,(13,(0,(2,(1,3))))),(12,((10,(9,11)),(5,(8,(6,7)))))))));
