# Supplementary material for: Genome-level homology and phylogeny of Vibrionaceae (Gammaproteobacteria: Vibrionales) with three new complete genome sequences
Source: BMC Microbiol. 2013 Apr 11;13:80. doi: 10.1186/1471-2180-13-80 (PMC3663674; doi:10.1186/1471-2180-13-80)
Supplement: Additional file 5 — Table S4. Genes Found in RAST Subsystems for All Species Part 2. [file 1471-2180-13-80-S5.pdf]

Table S4: Genes Found in RAST Subsystems for All Species Part 2:

(L = *V. brasiliensis*, M = *V. cholerae* ElTor, N = *V. corallillyticus*, O = *S. costicola*, P = *V. furnissii*, Q = *V. campbellii*, R = *G. hollisae*, S = *V. ichthyoenteri*, T = *V. metschnikovii*, U = *V. mimicus* M, V = *V. nigripulchritudo*).

RAST Subsystem	# in Fig. 7	L	M	N	O	P	Q	R	S	T	U	V
Cofactors, Vitamins, Prosthetic Groups, Pigments	1	307	297	325	373	314	325	288	299	256	289	372
Cell Wall and Capsule	2	159	201	198	251	175	181	177	183	145	221	189
Virulence, Disease and Defense	3	89	86	102	110	82	101	67	92	67	72	91
Potassium Metabolism	4	28	23	27	27	28	32	27	25	26	24	28
Photosynthesis		0	0	0	0	0	0	0	0	0	0	0
Miscellaneous	5	199	172	168	288	183	238	154	152	152	168	172
Phages, Prophages, Transposable elements, Plasmids	6	7	25	4	5	9	23	10	13	3	7	4
Membrane Transport	7	180	169	194	133	196	187	132	122	127	187	279
Iron acquisition and metabolism	8	56	47	73	23	56	60	50	37	36	60	43
RNA Metabolism	9	206	277	218	338	212	235	199	207	193	207	216
Nucleosides and Nucleotides	10	108	101	106	165	116	117	101	109	93	104	117
Protein Metabolism	11	226	227	242	337	229	260	240	187	206	229	244
Cell Division and Cell Cycle	12	33	41	36	61	34	34	36	38	34	32	31
Motility and Chemotaxis	13	181	141	194	204	222	172	158	113	117	182	151
Regulation and Cell signaling	14	109	107	124	158	105	121	70	114	88	104	129
Secondary Metabolism		0	3	0	0	16	0	0	0	3	0	3
DNA Metabolism	15	142	138	142	255	145	155	135	148	150	130	149
Fatty Acids, Lipids and Isoprenoids	16	129	102	121	130	125	107	106	106	90	99	174
Nitrogen Metabolism	17	53	22	54	77	38	38	16	28	13	33	43
Dormancy and Sporulation	18	4	2	6	6	3	4	2	3	3	2	4
Respiration	19	109	118	126	173	170	152	128	107	95	108	138
Stress Response	20	164	137	161	246	161	175	138	145	131	147	178
Metabolism of Aromatic Compounds	21	11	17	13	6	11	11	16	11	9	26	48
Amino Acids and Derivatives	22	477	339	467	537	471	425	340	344	349	366	420
Sulfur Metabolism	23	25	30	31	25	29	27	24	28	32	32	37
Phosphorus Metabolism	24	54	44	61	77	57	48	62	47	40	46	63
Carbohydrates	25	414	416	475	561	462	441	389	433	388	473	682
RNAs	26	61	123	121	196	132	153	125	66	112	119	86
coding sequences		4275	3632	5127	5575	4488	5325	3600	4432	3404	3897	5700
