# Supplementary material for: Genome-level homology and phylogeny of Vibrionaceae (Gammaproteobacteria: Vibrionales) with three new complete genome sequences
Source: BMC Microbiol. 2013 Apr 11;13:80. doi: 10.1186/1471-2180-13-80 (PMC3663674; doi:10.1186/1471-2180-13-80)
Supplement: Additional file 6 — Table S5. Genes Found in RAST Subsystems for All Species Part 3. [file 1471-2180-13-80-S6.pdf]

Table S5: Genes Found in RAST Subsystems for All Species Part 3:

W = *V. orientalis*, X = *V. parahaemolyticus*, Y = *V. scophthalmi*, Z = *V. mediterranei*, AA = *V. sinaloensis*, BB = *V. splendidus*, CC = *V.* sp. EJY3, DD = *V.* sp. Ex25, EE = *V.* sp. N418, FF = *V.* sp. RC341, GG = *V.* sp. RC586, HH = *V. tubiashii*, II = *V. vulnificus* CMC)).

RAST Subsystem	# in Fig. 7	W	X	Y	Z	AA	BB	CC	DD	EE	FF	GG	HH	II
Cofactors, Vitamins, Prosthetic Groups, Pigments	1	302	243	296	270	320	260	341	248	312	277	289	324	311
Cell Wall and Capsule	2	169	175	183	206	214	202	178	157	172	214	197	189	192
Virulence, Disease and Defense	3	89	81	88	111	87	89	82	81	88	62	67	111	85
Potassium Metabolism	4	28	25	26	32	29	19	33	29	26	25	26	24	24
Photosynthesis		0	0	0	0	0	0	0	0	0	0	0	0	0
Miscellaneous	5	180	133	169	186	202	191	261	160	161	122	156	189	218
Phages, Prophages, Transposable elements, Plasmids	6	19	8	23	10	4	19	6	11	4	4	5	10	9
Membrane Transport	7	189	189	178	211	199	193	194	181	148	146	155	262	209
Iron acquisition and metabolism	8	55	44	39	69	56	65	50	52	39	49	31	53	53
RNA Metabolism	9	206	196	203	212	211	225	215	192	203	195	198	216	296
Nucleosides and Nucleotides	10	108	100	104	128	117	101	115	85	109	99	95	123	110
Protein Metabolism	11	228	137	215	166	217	137	227	162	215	132	228	240	263
Cell Division and Cell Cycle	12	33	32	37	30	32	32	42	41	33	32	32	41	42
Motility and Chemotaxis	13	125	137	112	196	147	112	107	172	113	139	129	137	142
Regulation and Cell signaling	14	113	109	111	96	121	108	123	113	105	91	96	96	123
Secondary Metabolism		0	0	0	0	0	0	19	0	0	0	3	0	0
DNA Metabolism	15	129	139	141	109	136	114	138	129	123	153	143	156	141
Fatty Acids, Lipids and Isoprenoids	16	144	100	108	135	132	115	124	90	111	64	100	146	128
Nitrogen Metabolism	17	63	43	40	64	54	56	62	50	37	33	33	64	52
Dormancy and Sporulation	18	3	1	4	3	4	2	5	3	3	3	3	5	4
Respiration	19	118	114	124	145	122	149	147	141	113	106	109	114	113
Stress Response	20	160	148	147	172	168	147	195	169	147	135	140	168	168
Metabolism of Aromatic Compounds	21	12	11	10	36	11	20	37	9	13	12	9	13	9
Amino Acids and Derivatives	22	449	398	348	515	454	339	520	425	346	331	346	452	398
Sulfur Metabolism	23	29	12	26	46	32	43	43	27	27	27	29	36	44
Phosphorus Metabolism	24	53	46	44	87	55	54	48	46	46	47	45	56	50
Carbohydrates	25	424	424	422	648	478	404	620	385	425	430	390	507	492
RNAs	26	127	104	69	77	57	106	148	105	72	91	115	79	139
coding sequences		4204	4047	4534	5222	4529	4714	4830	4287	3985	3620	3684	5096	4637
